# Tumor suppressors in Sox2-mediated lung cancers promote distinct cell-intrinsic and immunologic remodeling

**DOI:** 10.1172/jci.insight.171364

**Published:** 2025-05-06

**Authors:** Nisitha Sengottuvel, Kristina M. Whately, Jennifer L. Modliszewski, Rani S. Sellers, William D. Green, Weida Gong, Allison T. Woods, Eric W. Livingston, Katerina D. Fagan-Solis, Gabrielle Cannon, Lincy Edatt, Hong Yuan, Aaron C. Chack, Yazmin Sanchez, Katherine Zhou, Alyaa Dawoud, Jarred M. Green, Virginia Godfrey, J. Justin Milner, Gaorav P. Gupta, Chad V. Pecot

**Affiliations:** 1 Lineberger Comprehensive Cancer Center,; 2 Curriculum in Genetics and Molecular Biology,; 3 Department of Pathology and Laboratory Medicine,; 4 Curriculum in Cell Biology and Physiology,; 5 Biomedical Research Imaging Center,; 6 UNC Advanced Analytics Core,; 7 Department of Radiology, and; 8 Department of Microbiology and Immunology, University of North Carolina, Chapel Hill, North Carolina, USA.; 9 Department of Radiation Oncology and; 10 Department of Medicine, Division of Hematology/Oncology, University of North Carolina School of Medicine, Chapel Hill, North Carolina, USA.; 11 RNA Discovery Center, University of North Carolina at Chapel Hill, Chapel Hill, North Carolina, USA.

**Keywords:** Genetics, Oncology, Innate immunity, Mouse models

## Abstract

Non–small cell lung cancer (NSCLC) largely consists of lung squamous carcinoma (LUSC) and lung adenocarcinoma (LUAD). Alterations in the tumor protein p53 (*TP53*) and phosphatase and tensin homolog (*PTEN*) tumor suppressors are common in both subtypes, but their relationship with *SOX2* is poorly understood. We deleted *Trp53* or *Pten* in a C57BL/6 *Sox2^hi^* *Nkx2-1^–/–^* *Lkb1^–/–^* (SNL) genetic background and generated a highly metastatic LUSC cell line (LN2A; derived from a Sox2^hi^ mouse model, followed by *Trp53*, *Pten*, and cyclin dependent kinase inhibitor 2A [*Cdkn2a*] deletion). Histologic and single-cell RNA-Seq analyses corroborated that SNL mice developed mixed tumors with both LUAD and LUSC histopathology while SNL-Trp53 and SNL-Pten mice developed LUAD and LN2A tumors that retained LUSC morphology. Compared with SNL mice, additional loss of *Trp53* or *Pten* resulted in significantly reduced survival, increased tumor burden, and altered tumor mucin composition. We identified a subcluster of CD38^+^ tumor-associated inflammatory monocytes in the LN2A model that was significantly enriched for activation of the classical and alternative complement pathways. Complement factor B (*CFB*) is associated with poor survival in patients with LUSC, and we observed the LN2A model had significantly improved survival on a *Cfb^–/–^* background. Our findings demonstrate a cooperative role of *Trp53* and *Pten* tumor suppressors in *Sox2*-mediated NSCLC tumor progression, mucin production, and remodeling of the immune tumor microenvironment.

## Abstract 2

Cell intrinsic and non-cell autonomous effects of tumor suppressors on Sox2-driven lung cancer

## Introduction

Lung cancer affects an estimated 2.3 million people worldwide every year (1). While incidence rates in the United States have been dropping by 2% annually, it continues to be the second most common cancer and the leading cause of cancer deaths (1, 2). Lung cancer is broadly divided into small cell lung cancer and non–small cell lung cancer (NSCLC), the latter of which consists of approximately 85% of all lung cancer. The 2 major subtypes of NSCLC are lung adenocarcinoma (LUAD) and lung squamous carcinoma (LUSC). LUAD typically occurs in the distal lung arising from alveolar type 2 epithelial cells or cells with bronchioalveolar duct junctions (3–5). These LUAD cells express characteristic markers, including transcription termination factor 1 (TTF1)/*NKX2-1* and keratin 7 (KRT7) (6). Conversely, LUSC typically occurs in the central airways arising from basal epithelial cells. LUSC classically appears as large polygonal cells with squamous differentiation expressing markers like TP63 and KRT5/6 (3, 5, 6). Oncogenic drivers are well characterized in LUAD, some of which include KRAS, EGFR, HER2, and MET mutations as well as oncogene fusions such as ROS1, ALK, NTRK, and RET, many of which already have approved targeted therapies that result in significantly improved survival (4, 7). However, thus far there are no analogous oncogenic drivers in LUSC that have proven to be clinically actionable. *SOX2* is located on the 3q amplicon, which is frequently amplified in LUSC, and has been found to be a bona fide LUSC oncogenic driver cooperating with *NKX2-1*, *LKB1*, or protein kinase C iota to promote a squamous differentiation (8–11). Although alterations of tumor suppressors such as tumor protein p53 (*TP53*), phosphatase and tensin homolog (*PTEN*), cyclin dependent kinase inhibitor 2A (*CDKN2A*), and Kelch-like ECH-associated protein 1 (KEAP1) are common in both LUAD and LUSC subtypes, their importance in SOX2-mediated NSCLC is largely unknown (12–14). Detailed understanding of these tumor suppressors’ roles both intrinsically on cancer cell biology and extrinsically in the tumor microenvironment (TME) of NSCLC is crucial in pursuing effective new treatment strategies.

Carcinogen-induced NSCLCs have high mutational burdens, and the use of genetically engineered mouse models (GEMMs) can help simplify complex pathways to determine which events are responsible for driving these cancers (4). The majority of NSCLC GEMMs that result in LUAD tumors have activating KRAS mutations (15). However, SOX2 overexpression in mouse club cells led to the formation of adenocarcinomas expressing the TP63 squamous marker (16). Combining SOX2 overexpression with common tumor suppressors PTEN and CDKN2A leads to LUSC-like tumors in basal, alveolar type 2, and club cells (17). Mouse models with *Sox2* overexpression combined with *Nkx2-1* and *Lkb1* loss (SNL model) demonstrated enhanced adeno-to-squamous differentiation of tumors as well as tumor-associated neutrophil (TAN) recruitment (11).

Utilizing a Lenti-Cre-Cas9–single guide RNA (sgRNA) approach for targeted deletion in the respiratory epithelium, we evaluated the impact of *Trp53* and *Pten* tumor suppressor deletions in *Sox2*-mediated NSCLC on tumor progression, TME composition, and tumor cell differentiation. Additionally, using a *Sox2*-driven LUSC model that grows in C57BL/6 mice (18), we used in vivo selective pressure to generate a potentially novel, highly metastatic subclone (LN2A). As compared with the autochthonous models, we found the LN2A model had markedly reduced survival because of metastatic progression, which was associated with recruitment of both tumor-associated inflammatory monocytes (TIMs) and macrophages (TAMs). We identified a subcluster of CD38^+^ TIMs in the LN2A model, which had enriched expression of genes associated with the activation of both the classical and alternative complement pathways. It has been established that classical complement activation in the TME results in enhanced tumor growth and increased metastasis (19), but in our study, we highlight that the alternative complement mediator, complement factor B (*Cfb*), may have a previously unappreciated role in LUSC metastases. This work reveals several cancer cell–intrinsic, histologic, and TME alterations that take place upon loss of *Trp53* or *Pten* tumor suppressors in *Sox2*-driven NSCLC.

## Results

### Loss of Trp53 or Pten in Sox2^hi^ GEMMs accelerates NSCLC progression.

**Figure 1:**
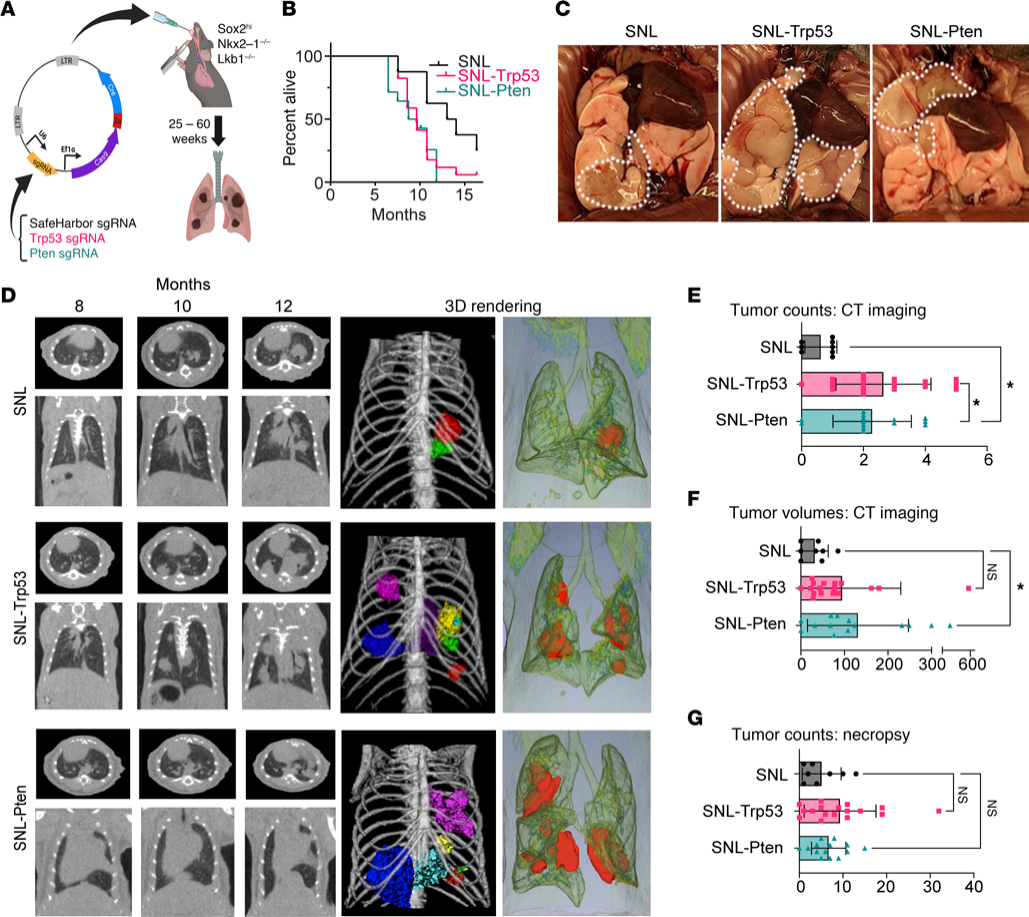
sgRNA-mediated CRISPR knockout of *Trp53* and *Pten* in vivo leads to increased tumorigenesis and decreased survival in Sox2^hi^ mice. (**A**) CRISPR-mediated knockout mice were created through tracheal instillation of LentiCRISPRv2Cre plasmid containing Safe Harbor (SH), *Trp53*, or *Pten* sgRNAs (10^5^ transfection units, TU). Virus was delivered into the lungs of mice. Mice (*n* = 8–17 per group) were monitored for up to 60 weeks and sacrificed upon tumor formation of greater than 5 mm. (**B**) SNL-Trp53 and SNL-Pten mice have significantly shorter survival times compared with SNL control mice (log-rank SNL-Trp53: *P* = 0.035, SNL-Pten *P* = 0.014, SNL *n* = 5, SNL-Trp53 *n* = 16, SNL-Pten *n* = 10). (**C**) Gross pathology images showing tumor burden (white outline) in SNL, SNL-Trp53, and SNL-Pten mice. (**D**) Representative imaging from coronal and axial views of CT scans showing disease burden over 8, 10, and 12 months for SNL and SNL-Trp53 mice and at 8, 10, and 11 months for SNL-Pten mice. Maximal disease burden highlighted with 3D tumor rendering images (right). (**E**) Total tumor counts from CT imaging (1-way ANOVA SNL-Trp53: *P* = 0.0016, SNL-Pten *P* = 0.0118). (**F**) Total tumor volumes by CT imaging. Volumes were calculated by measuring CT length × width^2^/2 (mm^3^). (1-way ANOVA SNL-Trp53: *P* = 0.2889, SNL-Pten *P* = 0.0276.) (**G**) Total tumor counts from gross pathology (1-way ANOVA SNL-Trp53: *P* = 0.2025, SNL-Pten *P* = 0.7716). (**E**–**G**) Data shown as mean ± SEM.

To evaluate the effects of 2 of the most common LUSC tumor suppressors — TRP53 and PTEN (Supplemental Figure 1; supplemental material available online with this article; https://doi.org/10.1172/jci.insight.171364DS1) — in a *Sox2^hi^* model of NSCLC (11), we intratracheally instilled sgRNAs in a LentiCRISPRv2Cre vector into SNL mice (11, 20) (Figure 1A). We used a lentiviral titer of 10^5^ to reduce the number of primary tumors and increase the survival of mice for longitudinal evaluation of tumor development (21). Compared with SH controls (SNL), which survived a median of 13 months, loss of *Trp53* (SNL-Trp53) or *Pten* (SNL-Pten) significantly decreased the median survival of these groups to 9 months (Figure 1B). The SNL-Trp53 and SNL-Pten mice had increased tumor counts, as visualized by gross pathology (Figure 1, C and G) and CT imaging (Figure 1, D–F). This shows that upon loss of *Trp53* or *Pten*, tumorigenesis latency decreases (Figure 1B) and tumor burden increases in the context of *Sox2* overexpression in the SNL mouse (Figure 1, E–G). Together, these findings demonstrate that loss of either *Trp53* or *Pten* tumor suppressor promotes SOX2-mediated NSCLCs.

### Development of a highly metastatic LUSC cell line.

**Figure 2:**
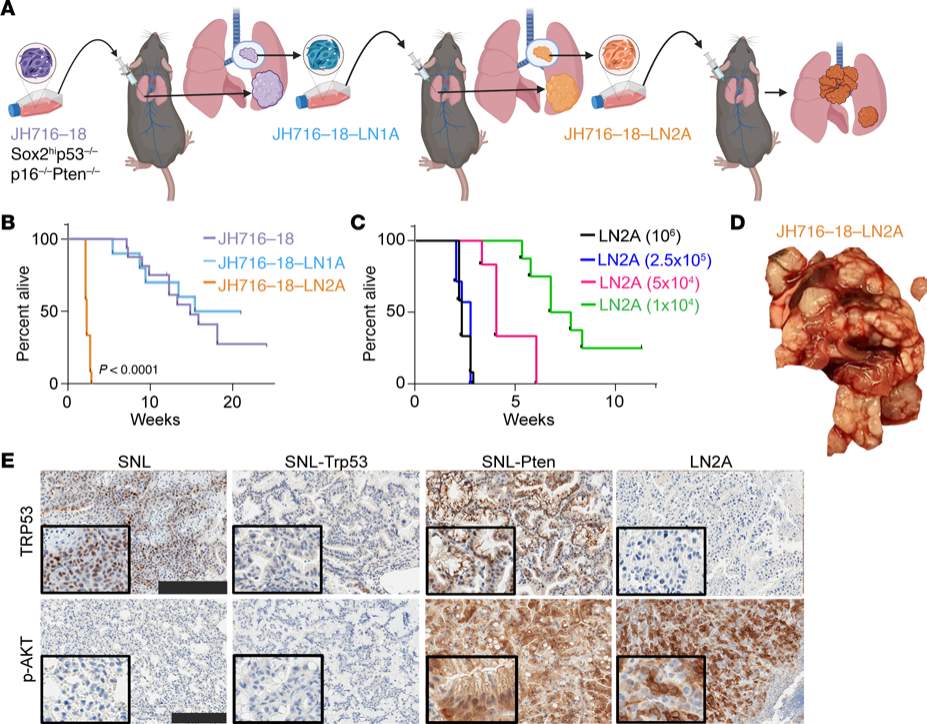
The development of the highly metastatic LN2A LUSC murine model. (**A**) In vivo selection was performed using JH716-18 LUSC cell line to create a metastatic LUSC cell line that could be injected into immune-competent C57BL/6 mice. The parental LUSC cell line, JH716-18, was injected into C57BL/6 mice. A lymph node tumor was collected at the time of necropsy and grown in vitro to create JH716-18-LN1A (LN1A). LN1A was then injected into C57BL/6 mice. A lymph node tumor was collected at the time of necropsy and grown in vitro to create JH716-18 LN2A (LN2A). (**B**) When injected into mice, LN2A reveals a highly metastatic phenotype with significantly decreased survival (log-rank JH716-18-LN1A: *P* = 0.438, LN2A *P* < 0.0001) compared with the parental JH716-18 and a prior subclone, LN1A. (*N* = 10–16 /group.) (**C**) LN2A remains highly metastatic even when lowering the number of cells injected (log-rank *P* < 0.0001) (*N* = 6–11/group). (**D**) Representative gross necropsy images from LN2A. (**E**) IHC-DAB of lung tumors from SNL, SNL-Trp53, SNL-Pten, and LN2A mice for TRP53 staining and p-AKT staining (scale bar = 200 μm).

To further evaluate the effect of *Trp53* and *Pten* mutations in SOX2-mediated NSCLC progression, we obtained the LUSC JH716-18 parental line and further created a highly metastatic cell line, LN2A. This cell line was derived from airway epithelial cell organoids from *Sox2*^hi^ Cas9 mice (C57BL/6 background) (18). Epithelial cells were harvested from murine trachea and lung and maintained as organoids in vitro and were subsequently treated with sgRNAs to knock out *Trp53*, *Cdkn2a*, and *Pten* expression, eventually resulting in the JH716-18 cell line (18). Using previously described methods (22, 23), we performed several rounds of in vivo selection by injecting the less metastatic JH716-18 orthotopically into mouse lungs, collecting lymph node metastases, and reinjecting the harvested and cultured cells until we isolated a highly metastatic LUSC subclone, which grows in immune-competent C57BL/6 mice (Figure 2A). When 10^6^ cells were injected orthotopically into the lungs of C57BL/6 mice, the parental JH716-18 line and first subclone, JH716-18-LN1A (LN1A), had median survivals of 15 and 18 weeks, respectively, and mice frequently succumbed to primary tumor growth. However, a subsequently isolated subclone, JH716-18-LN2A (LN2A), had a median survival of a little over 2 weeks (Figure 2B). The rapid progression of disease with LN2A allowed us to evaluate lower inoculation titers, and we observed metastatic progression with as few as 10^4^ cells (Figure 2C). For all subsequent experiments, we injected 10^5^ cells for the most consistent and reproducible disease progression. The tumorigenesis rate for the parental JH716-18 line was 70%, with slow growth and metastasis. However, LN2A had a 100% tumorigenesis rate with high metastatic proficiency, including more than 80% of mice having lymph node and bilateral chest wall metastases (Figure 2D and Table 1). To assess differences between LN2A and its parental cell lines (JH716-18 and LN1A), we performed bulk RNA-Seq on all 3 lines. Similar to their in vivo characteristics, the transcriptome analysis revealed JH716-18 and LN1A closely resembled each other, whereas LN2A exhibited distinct characteristics (Supplemental Figure 2A). Gene set enrichment analysis of the 3 cell lines revealed enrichment of mTOR complex 1 (mTORC1) signaling, MYC activation, and the unfolded protein response pathways in LN2A (Supplemental Figure 2B). It is known that about 70% of LUSC has aberrant activation of the mTOR pathway, leading to altered metabolism, increased proliferation, protein synthesis, evasion of apoptosis, and cell migration and invasion (12, 24, 25). The unfolded protein response pathway is used for cellular survival and has been established to contribute to cellular transformation, and promote proliferation, allowing for hypoxia and nutrient deprivation and suppression of antitumor immune responses (26). Our findings suggest that our in vivo passaging led to accelerated disease progression and activation of mTORC1, MYC, and unfolded protein response pathways. The highly metastatic LN2A LUSC model allowed us to study the aggregate roles of the 3 most common tumor suppressors in LUSC (*Trp53*, *Pten*, and *Cdkn2a*, Supplemental Figure 1) in a C57BL/6 immune-competent background. This model demonstrates the importance of *Trp53*, *Pten*, and *Cdkn2a* for the development of metastatic LUSC and will serve as a valuable model for the NSCLC field.

### SNL models demonstrate NSCLC transdifferentiation.

**Figure 3:**
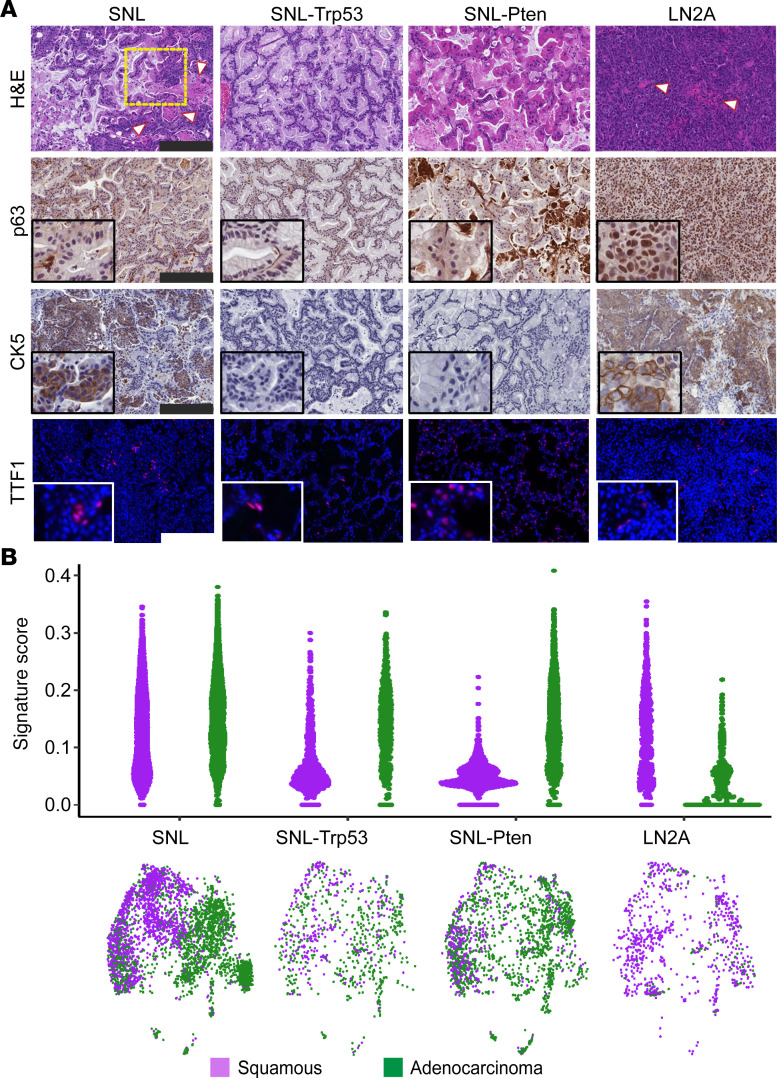
Genetic regulation of squamous or adenocarcinoma transdifferentiation of NSCLC tumors. (**A**) Histology was performed on FFPE tumor tissue sections of the lung tumor (SNL, SNL-Trp53, SNL-Pten) or LN tumors (LN2A). Tissues were stained with H&E, squamous markers p63 and CK5, and adenocarcinoma marker TTF1, red (DAPI, blue). White arrowheads = keratin deposits. Yellow box = region of adenomatous and squamous histologies mixing. Scale bar = 200 μm. (**B**) Sina plots of adenocarcinoma and squamous signature scores for all tumor cells by model. Wilcoxon rank-sum tests show the difference between scores for all cells within each model. Uniform manifold approximation and projection (UMAP) of all tumor cells (*n* = 6,808 cells) colored by adenocarcinoma or squamous classification; cells were classified as adenocarcinoma when their score was higher for adenocarcinoma than squamous. UMAP is split by model (SNL = 3,418 cells, SNL-Trp53 = 768 cells, SNL-Pten = 1,828 cells, LN2A = 794 cells).

Immunohistochemistry (IHC) staining for TRP53 and phosphorylated Akt (p-AKT), a marker of PTEN deletion, verified Trp53 and Pten loss in SNL-Trp53 and SNL-Pten groups, respectively, and dual Trp53 and Pten loss in LN2A tumors (Figure 2E). Two board-certified veterinary pathologists independently evaluated H&E-stained tumors in a masked fashion. Lung tumors in the SNL model were a mixture of both LUSC and LUAD tumor types. Observed LUSC features included squamous differentiation of tumor cells, often associated with keratinization (keratin pearls). LUAD features included tumor islets with goblet cells characteristic of adenocarcinomas. In contrast, lung tumors in SNL-Trp53 and SNL-Pten mice were only LUAD tumor type. The LN2A orthotopic model maintained a pure LUSC morphology (Figure 3A). The LUSC histopathologic observations were corroborated with IHC markers used to clinically differentiate LUAD and LUSC in humans (27). Consistent with the mixed histologic findings, tumors from SNL mice had positive staining for CK5 (LUSC marker), while the SNL-Trp53 and SNL-Pten mice lacked CK5 and nuclear p63 (LUSC markers) (Figure 3A). The tumor samples with isolated TTF1 positivity were reflective of the presence of normal alveolar cells in the lungs, rather than positive tumor staining (Supplemental Figures 3 and 4). Surfactant protein C (SPC) positivity identified normal angiotensin type 2 cells within the normal lung tissue and surrounding the tumors, with few instances of SPC positivity within the tumors (Supplemental Figure 5A). LN2A mice expressed p63 and CK5 LUSC markers (Figure 3A). To further characterize these models, we performed single-cell RNA-Seq (scRNA-Seq) analysis on tumors from each group (SNL, SNL-Trp53, SNL-Pten, and LN2A, *n* = 4 mice per group), sequencing a total of 61,529 cells that passed quality control. We defined each cancer cell within the tumor cell clusters as either squamous or adenocarcinoma based on previous signatures (28) (Supplemental Table 1 and Supplemental Figures 6 and 7). Consistent with our morphologic and IHC findings of mixed histologies, while SNL tumor cells had higher adenocarcinoma signature scores than squamous signatures, a portion of tumor cells had an elevated squamous signature. The SNL-Trp53 and SNL-Pten groups had significantly higher adenocarcinoma signature scores than squamous, and the LN2A tumor cell clusters had a significantly elevated squamous signature (Figure 3B), all of which are consistent with our histologic observations. Unlike the LN2A model, a notable distinction in the SNL models is the genetic removal of the *Lkb1* tumor suppressor, which has previously been linked to histologic transdifferentiation and increased H3K27 methyltransferase activity in the LUSC cells via EZH2 expression (29). The genetic context of these NSCLC models helps inform the differentiation of the tumors into either LUAD or LUSC.

### Pten loss alters the mucinous differentiation of lung tumors in SNL mice.

**Figure 4:**
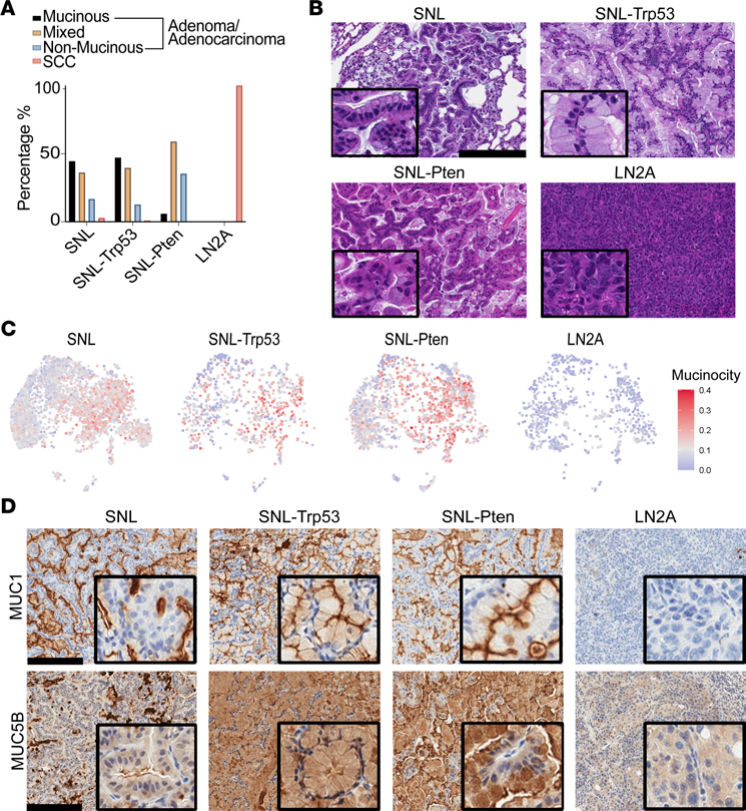
Pten loss alters LUAD mucin composition and affects lipid pathways in the TME. (**A**) Percentage of mucinous, nonmucinous, or mixed histology of tumors from all models as scored by a veterinary pathologist using FFPE H&E-stained slides from lung tumors (SNL models) and LN tumors (LN2A); χ^2^ (4, *N* = 187) = 22.12; *P* < 0.001. Atypical adenomatous hyperplasias, adenomas, and adenocarcinomas were combined for the purposes of this work (SNL *n* = 6 mice, SNL-Trp53 *n* = 13 mice, SNL-Pten *n* = 8 mice, and LN2A *n* = 12 mice). SCC, squamous cell carcinoma. (**B**) Representative H&E images showing mucinous and nonmucinous histologies found in the LN2A and SNL models. (**C**) UMAPs of tumor cells showing gene signature score driving mucinous adenocarcinoma. (**D**) IHC-DAB of lung tumors from SNL, SNL-Trp53, SNL-Pten, and LN2A mice for MUC1 and MUC5B staining (scale bar = 200 μm).

LUAD tumors can differentiate into mucinous and/or nonmucinous adenocarcinomas, with the nonmucinous subtype comprising the majority of LUAD cases (30). In our models, we observed that the SNL and SNL-Trp53 tumors contained almost equal amounts of mucinous and mixed (mucinous/nonmucinous) components, along with a small percentage of nonmucinous and squamous histology. Interestingly, the adenocarcinomas from the SNL-Pten mice had the highest percentage of mixed (mucinous/nonmucinous) components with few purely mucinous regions, which was notably different from the tumors in SNL and SNL-Trp53 mice (Figure 4, A and B). A χ^2^ test demonstrated that the proportion of tumors classified as mucinous, mixed, or nonmucinous was statistically different between the different SNL models (χ^2^ = 22.12, *P* < 0.001). To determine whether these tumors are indeed a mix of LUSC and LUAD cells, or perhaps biphenotypic (with both LUSC and LUAD markers expressed in the same populations of cells), we used our transcriptional profiling to compare dual expression of LUSC and LUAD markers by identifying *p63*^+^*CK5*^+^ tumor cells and evaluating them for hepatocyte nuclear factor 4 alpha (*Hnf4a*) expression. The majority of *Hnf4a*-positive cells were found in *p63*- and *CK5*-negative cells across all SNL groups, consistent with the LUAD cells producing a mucinous state. A negligible number of *Hnf4a*^+^ cells were dual or triple positive with *p63* and *CK5* (Supplemental Figure 8 and Supplemental Figure 5B). When mucin-high cells were compared between SNL-Pten and SNL, SNL-Pten and SNL-Trp53, and SNL-Trp53 and SNL, in separate analyses of differential expression, we saw very few differences in the mucin-high cells among models. We observed that the SNL-Pten group had the most balance between the number of mucin-high and mucin-low tumor cells, and SNL-Trp53 had the highest mucin signature scores (Figure 4C and Supplemental Figure 9). SNL, SNL-Trp53, and SNL-Pten tumors exhibited regions of MUC1-positive staining, indicating mucinous differentiation, interspersed with regions lacking MUC1 staining, reflective of distinct mucinous and nonmucinous areas within the tumors. MUC5B expression was absent in the SNL model but markedly increased in SNL-Trp53 and SNL-Pten tumors (Figure 4D). Notably, SNL-Pten tumors displayed more sharply defined regions of MUC5B positivity adjacent to MUC5B-negative areas, highlighting possible spatial heterogeneity within these tumors. As expected, LN2A was negative for MUC1 and MUC5B staining (Figure 4D). Evaluation of UMAPs and correlation of our tumor cells for mucinous and proliferative signatures showed a lack of overlap (Supplemental Figure 10), suggesting that the mucinous differentiation in the adenocarcinoma cells is not a result of increased proliferative programming in the tumor cells. A gene set variation analysis (GSVA) testing differences in mucin-high and mucin-low cells within the LN2A and 3 SNL models revealed that the Reactome HDL assembly gene signature was upregulated significantly only in the SNL-Pten comparison (Supplemental Table 2). To evaluate whether patients with LUSC or LUAD have differences in mucin or HDL signature expressions based on their *PTEN* status, we performed a GSVA of The Cancer Genome Atlas (TCGA) LUSC and LUAD patients with and without altered *PTEN* (LUAD patients *n* = 504, LUSC patients *n* = 466). Interestingly, LUSC tumors with *PTEN* alteration had significantly higher mucinous GSVA scores when compared with unaltered *PTEN* (*P* = 0.032) (Supplemental Figure 11A). We also found that HDL assembly signatures did not significantly correlate to *PTEN* alteration (Supplemental Figure 11B). The lack of correlation of HDL assembly signatures and *PTEN* alteration suggests that the *PTEN*-driven mucinous changes we observe may not be driven by the HDL pathway in humans. Collectively, our findings demonstrate that *Pten* loss drives substantial alterations in the mucinous composition of SNL model tumors, which was corroborated by clinical TCGA LUSC data demonstrating increased mucinous gene signatures in tumors with *PTEN* alterations.

### LUSC tumors recruit Ly6C^+^ inflammatory monocytes and CCR5^+^ macrophages.

**Figure 5:**
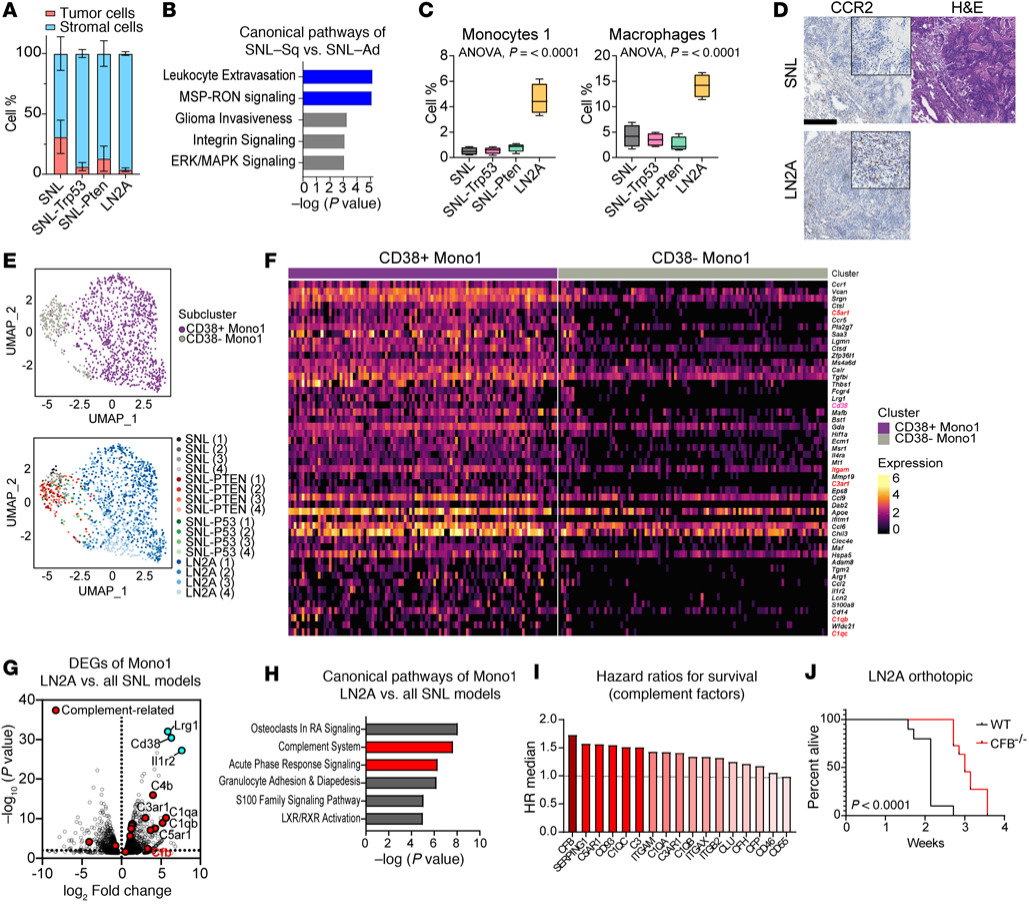
Increased recruitment of inflammatory myeloid subsets in squamous tumors. (**A**) Tumor and stromal cell composition as defined by scRNA-Seq clustering in all models. (**B**) QIAGEN’s Ingenuity Pathway Analysis (IPA) was performed on differentially expressed genes (DEGs) identified between squamous-aligned (SNL-Sq) and adenocarcinoma-aligned (SNL-Ad) tumor cells from SNL mice. Pathways shown are enriched in squamous-aligned tumor cells. Blue: immune cell recruitment pathway. (**C**) Percentage of Mono1 and Mac1 in models. Box plots show the interquartile range, median (line), and minimum and maximum (whiskers). (**D**) IHC staining for CCR2 in SNL and LN2A tumors (scale bar = 200 μm). (**E**) UMAP plots showing subclustering of Mono1 subpopulation (*n* = 1,235 cells) by cluster and by experimental group. (**F**) Heatmap showing DEGs by clusters, shown in **E**. (**G**) DEGs of Mono1 in LN2A versus all SNL models. Aqua blue: most highly differentially expressed. Red: complement related. (**H**) IPA of DEGs to get enriched canonical pathways of LN2A Mono1 versus Mono1 from all SNL models. Red: complement related. (**I**) Hazard ratios (HR median) for survival relevance of complement factors. (**J**) A total of 100,000 LN2A cells were injected orthotopically into the left lungs of either wild-type or *Cfb*^–/–^ mice (*n* = 10/group) (log-rank *P* < 0.0001).

We observed that as the models became increasingly aggressive, the proportion of cancer cells in the tumors decreased, while immunologic proportions increased. While the SNL tumors had the highest proportion of cancer cells (31%), the SNL-Trp53 and SNL-Pten tumors had 6% and 13% cancer cells, respectively, and the LN2A tumors consisted of only 4% cancer cells by scRNA-Seq counts (Figure 5A). The lower cancer cell count in the LN2A samples may in part be explained by technical biases in scRNA-Seq, such as loss of sample due to necrosis of tumor cells, or the immune-rich environment of lymph node metastases, leading to increased infiltration of smaller sized immune cells, as illustrated by CD45 and pan-cytokeratin staining (Supplemental Figure 12). Because the SNL model had a substantial number of squamous-aligned (SNL-Sq) and adenocarcinoma (SNL-Ad) cells, it created an opportunity to study differences between transdifferentiated tumor cells in the same genetic context. In GSVA of SNL-Sq versus SNL-Ad cells, we observed that the SNL-Sq tumor cells closely matched that of the LN2A tumor cells, whereas the SNL-Ad tumor cells matched that of the SNL-Trp53 and SNL-Pten tumor cells (Supplemental Figure 13). When conducting a canonical pathway analysis on the differentially expressed genes (DEGs) (Supplemental Table 3 and Supplemental Figure 14) in SNL cancer cells through Ingenuity Pathway Analysis (IPA; QIAGEN), inflammatory immune recruiting pathways such as the MSP-Ron signaling in cancer cells pathway and leukocyte extravasation signaling were enriched in SNL-Sq cells (Figure 5B). This opportunity to examine transdifferentiated squamous and adenocarcinoma tumors on the same genetic background allowed us to uncover the increased immune trafficking present in LUSC tumors.

There were several variations between the composition of the immune microenvironment between the 4 groups (Supplemental Figure 15) by scRNA-Seq. Notably, SNL had far fewer B cells than any other group, with LN2A having the highest B cell population. Loss of *Trp53* led to increases in TANs with no increase of neutrophil subsets commonly found in healthy lungs (31). Specifically, neutrophil N3-N5 subsets were elevated in the SNL-Trp53 and LN2A groups (Supplemental Figure 16). Even compared with LN2A, the SNL-Trp53 mice had the highest levels of “neutrophil 5” *Gpnmb^+^* inflammatory neutrophil percentage (Supplemental Figure 15), which aligns with previous work showing upregulation of *Gpnmb* expression and neutrophilia by *Trp53* loss (32, 33). LN2A also had far fewer endothelial cells than the other groups, and dendritic cells (DCs) made up a small minority of the population across all the groups. Though not significant, X-C motif chemokine receptor 1–positive (XCR1^+^) DC1s were scarcer in the LN2A group than in the SNL groups (Supplemental Figure 15). XCR1^+^ DC1s are key enhancers of antitumor responses, so their reduced numbers may have played a role in creating a more permissive TME for LN2A’s aggressive growth (34, 35). Together, we found several changes in immune composition driven by tumor genotype that link to increasingly aggressive tumor progression.

### CD38^+^ TIMs associate with complement pathways in the LN2A model.

One of the largest differences observed as tumors progressively became more aggressive is the significant increase in levels of “Mac1,” *Ccr5*^+^ inflammatory TAMs, and “Mono1,” LY6C^hi^ inflammatory TIMs, most notably in the LN2A model (Figure 5C). IHC staining with CCR2, a marker of inflammatory monocytes, verified these findings, showing markedly increased CCR2^+^ stained cells in tumors from the LN2A model compared with squamous histologic regions from SNL mice (Figure 5D) (23). By comparing Mono1/TIM clusters across the tumors from the 4 groups, we observed 2 distinct subclusters: CD38^+^ TIMs and CD38^–^ TIMs (Figure 5E). The composition of Mono1 in LN2A almost solely consisted of CD38^+^ TIMs, while the CD38^–^ TIMs were largely enriched in all 3 SNL models. To assess differences in CD38^+^ TIMs across genetic models, we used multiplex immunofluorescence to identify dual CCR2^+^CD38^+^ cells. LN2A tumors showed significantly higher numbers of dual-positive cells compared with the SNL and SNL-Pten groups (SNL *P* = 0.036 and SNL-Pten *P* = 0.019) and a nonsignificant increase compared with the SNL-Trp53 group (*P* = 0.369) (Supplemental Figure 17). By comparing the markers between these 2 TIM subclusters, we observed that several therapeutic targets were upregulated in CD38^+^ TIMs, such as CCR1 and CCR5, and we noticed significant increases in complement pathway–related molecules (Figure 5F and Supplemental Table 4). Differential expression analysis of TIMs from LN2A model compared with the SNL models revealed highly significant increases of leucine-rich α-2 glycoprotein 1 (*Lrg1*) and interleukin-1 receptor type 2 (*Il-1r2*) upregulation in addition to CD38 (Figure 5G). LRG1 is an adipokine that has been shown to promote angiogenesis and promote immunotherapy resistance in cancers (36–40). IL-1R2 is a nonsignaling receptor that serves as a decoy to limit activity of its binding partners: IL-1A, IL-1Β, and IL-1R (41). Interestingly, IL-1R2 has been found to be reduced in monocytes and macrophages in atherosclerotic lesions (42), whereas we find that the CD38^+^ TIM subclusters are enriched for IL-1R2. A CD38^+^ myeloid-derived suppressor cell (MDSC) population has been reported in pro-metastatic models and patients with advanced-stage head and neck and colon cancer (43, 44). However, little is known about whether CD38^+^ TIMs promote cancer progression. Consistent with a CD38^+^ TIM population in the LN2A model, upon CD38 depletion, we observed a statistically significant decrease in TIMs and monocytic MDSCs and a significant increase in T cells (Supplemental Figure 18A). However, despite these immunologic effects, CD38 depletion did not result in significant survival benefits (Supplemental Figure 18B). Also, in a cross-sectional study, CD38 depletion did not significantly reduce metastases (Supplemental Figure 18, C and D). However, aside from CD38^+^ TIMs, our single-cell data revealed that CD38 was expressed in several other myeloid populations in the LN2A model, and thus CD38 depletion likely has pleiotropic effects in the TME (Supplemental Figure 19A). Consistent with the depletion studies, we observed no association with global tumor CD38 expression and survival in the TCGA LUSC dataset (Supplemental Figure 19B). We evaluated MDSC and inflammatory monocyte signatures in LUSC TCGA data and observed no clear associations with *PTEN* or *TRP53* status in a *SOX2* context (Supplemental Figure 20). Evaluating the CD38^+^ TIM signature and a previously published TIM signature across LUAD and LUSC TCGA datasets, we observed a dynamic range in each NSCLC subtype with generally equal abundance of TIM subsets (Supplemental Figure 21A) (23). Furthermore, by evaluating scRNA-Seq data from published clinical LUSC and LUAD samples (23, 45), we observed major variability in the proportion of myeloid populations between LUSC and LUAD and increased proportions of all 3 monocyte subtypes in LUSC compared with LUAD (Supplemental Figure 21B).

Because we could not specifically deplete and evaluate the impact of CD38^+^ TIMs on metastases, we examined pathways that arose from DEGs between LN2A TIMs versus TIMs from the SNL groups, which revealed that complement activation may be involved in the promotion of LUSC progression (Figure 5H). Genes enriched in our dataset that belonged to the complement system included members of both classical and alternative pathway activation: *C4b*, *C1qa*, *C3ar1*, *C1qb*, *Itgam*, *Cfp*, *C1qc*, *Cd93*, *C5ar1*, *Cfh*, *Clu*, *C3*, and *Cfb* (Supplemental Table 5). Among these, *CFB* correlated with the worst hazard ratio for patient survival in LUSC TCGA data (Figure 5I). High *CFB* expression has previously been implicated in worse survival in thyroid carcinoma and LUAD (46, 47). Compared with wild-type mice orthotopically injected with the LN2A cells, we found that *Cfb*-knockout mice had significantly increased survival (Figure 5J, log-rank *P* < 0.0001), which was unrelated to a reduction in CCR2^+^CD38^+^ TIMs (Supplemental Figure 17A). Due to the known association between tumor genotype and programmed cell death 1/programmed cell death ligand 1 (PD-1/PD-L1) expression in the TME, we evaluated whether PD-1/PD-L1 expression varied across the 4 tumor models (48, 49). The pseudobulk differential expression analysis revealed a significant decrease in *Pdl1/Cd274* expression on macrophages and significant increase in *Pd1/Pdcd1* on fibroblasts in the LN2A model (Supplemental Figure 22). To specifically evaluate for any association between *CFB* expression and *PD/PDL1* expression in LUSC and LUAD patient samples, we performed a TCGA correlation analysis. While all comparisons demonstrated high levels of significance and positive correlation (*P* < 0.0001), the highest positive correlation was for LUSC *PD1* with *CFB* (Supplemental Figure 23). Taken together, these findings suggest that an alternative complement activation pathway via CFB may have previously unappreciated roles in LUSC progression.

## Discussion

TRP53 and PTEN are well-known tumor suppressors that are vital for NSCLC progression (50, 51). Many things are known about the cancer cell–intrinsic effects of aberrant TRP53, such as inducing genetic instability (52), accelerating proliferation (53–55), modulating metabolism (56, 57), promoting metastasis (58–60), and inducing chemo- or radioresistance (61, 62). Extrinsic effects of *TRP53* mutations include development of a pro-oncogenic TME via changes in cytokine expression, angiogenesis, and ECM remodeling (62–66). While loss of *TRP53* is common in both LUSC and LUAD, *PTEN* loss is more commonly associated with LUSC and identified in only about 3% of LUAD cases (51, 67). PTEN is a tumor suppressor that results in increased p-AKT signaling when lost or mutated, which results in cell-intrinsic effects on cell cycle progression, proliferation, chemotaxis, etc. (68). The majority of what is known about PTEN and TRP53 results from KRAS-mutant lung cancer models; thus, it is critical to better understand the effects of these tumor suppressors in the context of SOX2 overexpression, which may have greater relevance to patients with LUSC.

To evaluate the role of key tumor suppressors of *Trp53* and *Pten* in a genetic context relevant to Sox2-mediated lung cancer, we harnessed a potentially unique mouse model harboring floxed *Nkx2-1* and *Lkb1* deletions in the context of *Sox2* overexpression (11). *SOX2* is amplified in approximately 40% of all LUSC tumors (Supplemental Figure 1) and a bona fide oncogenic driver of LUSC (11). *LKB1* deletions are almost twice as common in LUAD patient tumors compared with LUSC tumors (69). However, *LKB1* loss is known to promote transdifferentiation to squamous tumors (70), and *Lkb1* deletion in the presence of *Sox2* promotes the formation of squamous tumors in mouse models (10, 11). The SNL models already have *Nkx2-1* loss and accelerated LUSC tumorigenesis (9, 11). Previous work has found that completely deleting *Nkx2-1* leads to a model that produces mucinous adenocarcinomas and adenosquamous and squamous cell carcinomas (11). We observed that while the SNL model resulted in mixed adeno-squamous histologies, the addition of *Trp53* and *Pten* deletion heavily skewed toward adenocarcinomas. It is important to note that tracheal instillations of sgRNA lentivirus leads to targeting the virus into the airway epithelium, which contains several types of epithelial cells, such as ciliated, columnar, undifferentiated, secretory, and basal epithelial cells (71).

In humans, LUAD is primarily nonmucinous; mucinous differentiation of these tumors is associated with worse survival (72). The SNL mouse models yielded LUAD-like tumors with either mixed mucinous (SNL-Pten) or mostly mucinous differentiation (SNL and SNL-Trp53). This differentiation may be attributed to the deletion of *Nkx2-1* in the SNL model (73). HDL assembly uniquely appearing as an enriched gene set for mucin-high samples only in the SNL-Pten group suggests that *Pten* loss in the SNL genetic context may lead to alteration of the TME mucin and lipid composition. These findings are consistent with PTEN’s negative regulation of PI3K signaling and lipid phosphatase activity (74, 75).

Previous studies have shown variations between the immune infiltrate found in LUSC compared with LUAD, such as LUAD having an increased percentage of resting memory CD4^+^ T cells and LUSC having increased percentages of plasma cells in the TME (76). Our work delves further into determining differences seen between LUAD and LUSC by looking at our SNL model, which we found contains both adenocarcinoma and squamous carcinoma tumor cells in the same genetic context. By evaluating DEGs between histologic subtypes, we found that macrophage signaling and leukocyte extravasation were significantly upregulated in LUSC cells (Figure 5B). The myeloid recruitment pathways in LUSC tumor cells were further supported by increased levels of multiple myeloid populations in the purely squamous LN2A model.

No metastatic LUSC mouse syngeneic models have been reported for use in C57BL/6 mice. By utilizing the LUSC syngeneic cell line, JH716-18 (18), we generated a highly metastatic subclone, LN2A, which allowed us to study metastatic squamous tumor progression in an immunocompetent C57BL/6 background. A limitation of our study with the SNL models is the lack of a *Cdkn2a*-knockout group, while the LN2A model contains triple loss of *Cdkn2a*, *Trp53*, and *Pten*. Also, the LN2A model requires orthotopic implantation and has more rapid disease progression. However, our integrated study of this model with the autochthonous lentivirus-induced GEMMs, along with scRNA-Seq analyses, resulted in the finding that more rapid disease progressions were strongly associated with robust recruitment of myeloid populations and signatures of unfolded protein response, MYC, and mTORC1 signaling pathways.

The LN2A model revealed several observations about the potential role of TIMs in LUSC progression, particularly regarding a CD38^+^ TIM subcluster, which may also have roles in various other cancers. Previous work has shown the existence of CD38^+^ MDSC populations in cancer (43, 44), and others have found that cancer cells can overexpress CD38 on their cell surface following development of acquired resistance to PD-1/PD-L1 blockade (77). Importantly, a phase II clinical trial in NSCLC using daratumumab, a monoclonal antibody that targets CD38, showed no improved efficacy in patients who received daratumumab and anti–PD-L1 compared with anti–PD-L1 monotherapy (78). These findings are consistent with our CD38 depletion studies in the LN2A model demonstrating no reduction in metastases or improvement in survival. An important caveat is that such depletion studies target all populations of CD38^+^ cells and do not specifically eliminate only CD38^+^ TIMs. However, gene ontology analyses revealed that CD38^+^ TIMs may be key activators of the classical and alternative complement cascades. In support of these pathways being involved in LUSC progression, we observed a highly significant survival increase in a *Cfb*^–/–^ mouse using the LN2A model and that high *CFB* in patients with LUSC was associated with significantly worse overall survival.

Here, we find several biologic effects of *Trp53* and *Pten* loss in *Sox2*-mediated NSCLC models, which include increased metastases, effects on histologic differentiation and mucin production, and notable changes in the immunologic TME. Also, the use of a *Sox2*-mediated, highly metastatic LUSC model revealed progressive increases in recruitment of myeloid subsets to the TME as the models became more metastatic. Finally, our finding that deletion of *Cfb* reduces disease progression warrants further investigation and may provide a new therapeutic vulnerability in the treatment of NSCLC.

## Methods

### Sex as a biological variable.

These studies included both male and female animals, and sex was largely not considered as a biological variable since lung cancer equally effects each sex.

### Gibson cloning and lentiviral production.

Sequences for sgRNA oligos for *TRP53* (79, 80), *PTEN* (81), and SH sites were flanked with 5′ (GTGGAAAGGACGAAACACCG) and 3′ (GTTTTAGAGCTAGAAATAGCAAGTTAAAATAAGG) adaptor regions and ordered from Eton Bioscience Inc (Supplemental Table 6). The oligos were PCR-amplified using a Q5 High-Fidelity DNA Polymerase PCR (New England BioLabs; NEB). The PCR product was then purified using the QIAGEN MinElute kit and eluted in elution buffer. The DNA was quantified using the Qubit fluorimeter and high-sensitivity DS DNA assay kit.

The LentiCRISPRv2Cre vector (20) was digested using the NEB 3.1 buffer and BsmBI overnight at 55°C before heat inactivation at 80°C for 20 minutes. A total of 100 ng of vector and 2 ng of insert were used in preparing NEB HiFi assembly reactions at a 1:2 molar ratio. The reaction was incubated for 50°C for 1 hour. The HiFi reaction was added to Endura electrocompetent cells on ice and then electroporated in a prechilled gap cuvette followed by recovery at 37°C for 1 hour. The cells were then plated on prewarmed Luria-Bertani broth Ampicillin Agar Plates.

HEK293T cells (ATCC) were transfected with PEI (Polysciences, 23966) and pEFGP-N2 to label transfection efficiency. Media containing virus were collected for 3 days, filtered, purified by ultracentrifugation at 22,000*g*, and evaluated for functional titer by flow cytometry (Thermo Fisher Attune NxT).

### Tracheal instillation.

Prepared LentiCRISPRv2Cre containing the designated sgRNAs (10^5^ TU) were delivered into the lungs of anesthetized R26-LSL-*Sox2*-GFP *Nkx2-1^fl/fl^* *Lkb1^fl/fl^* (SNL) mice (provided to us by Trudy Oliver, Duke University, Durham, North Carolina, USA) through orotracheal instillation in a final volume of 75 μL. Mice were monitored weekly after instillation and CT scanned monthly starting 7 months after instillation. Once scans showed abundance of tumor formation (>5 mm), mice were taken and sacrificed, and tumor tissues were collected for histology, bulk RNA-Seq, and scRNA-Seq. Due to availability from our breeding colony, both male and female mice were used in these studies. For sequencing there was an uneven distribution of sexes taken for sequencing (SNL: 1F, 3M; SNL-Trp53: 3F, 1M; SNL-Pten: 4F).

### Generation of JH716-18 subclones.

The JH716-18 cell line derived from CRISPR-mutant organoids of Sox2 Cas9 tracheal epithelial cells were obtained from the Wong Lab (New York University Langone, New York, New York, USA). These cells were then orthotopically injected into the left lung lobe of C57BL/6 mice and serially passaged in vivo to yield the JH716-18-LN1A cell line. JH18-LN1A cells were then serially passaged in C57BL/6 mice to generate a highly metastatic, aggressive subclone, JH716-18-LN2A cells (LN2A). The LN2A cell line was cultured in DMEM/F-12 media (Gibco 11320-033) supplemented with Penicillin-Streptomycin, 10% FBS, and Glutamax (Gibco 35050-061). They were washed with warm 0.25% Trypsin-EDTA for 5–10 minutes at 37°C, then incubated for another 10 minutes at 37°C in fresh 0.25% Trypsin-EDTA for passaging. All cells were maintained at 37°C in 5% CO_2_. For each orthotopic injection, cells were trypsinized, washed in saline solution, and resuspended in Hanks’ balanced salt solution (HBSS; Gibco) prior to injection of 1 × 10^6^ cells in a 1:1 mix of HBSS and Matrigel (Corning 356237). Female mice were used for these studies.

### Mouse orthotopic tumor model studies.

Adult C57BL/6 mice were purchased from The Jackson Laboratory, and C57BL/6-*Cfb*^–/–^ mice were obtained from the Wenchao Song (Perelman School of Medicine, Philadelphia, Pennsylvania, USA) and Rick Wetsel (UT Houston Texas Medical Center, Houston, Texas, USA) labs (82). Animals were 8–12 weeks of age at the time of tumor cell injection. LN2A cells were trypsinized, washed in PBS, and resuspended in HBSS prior to injection (*n* = 10/group). A total of 10^5^ LN2A cells were orthotopically injected into the left lungs of the mice in a 1:1 mix of HBSS and Matrigel at a final volume of 50 μL. Mice were monitored by weight twice weekly and euthanized upon exhibiting signs of morbidity (lethargy, dyspnea, 20% weight loss) due to tumor burden. For the CD38 depletion studies, isotype (Bio X Cell BE0089) and anti-CD38 (Bio X Cell BE0317) in vivo antibodies were used at 250 μg/mouse once per week.

### Histology.

Tissues were fixed in 10% neutral buffered formalin (Thermo Fisher Scientific) and processed routinely to paraffin. Sections were stained routinely by H&E. IHC was performed against TTF1, TRP53, p-AKT, MUC1, MUC5b, CCR2, CK5, CD45, SPC, and P63. IHC for TTF1, p53, p-AKT, MUC1, MUC5b, CD45, SPC, and CCR2 was performed on 5 μm paraffin sections using the Leica Biosystems Bond III Autostainer system. Slides were dewaxed in Bond Dewax solution (AR9222) and hydrated in Bond Wash solution (AR9590). Heat-induced antigen retrieval was performed at 100°C in either Bond-Epitope Retrieval solution 1 pH-6.0 (AR9961) or Bond-Epitope Retrieval solution 2 pH-9.0 (AR9640). The antigen retrieval was followed with a 5-minute peroxide blocking step (Biocare, IPB5000L). After pretreatment, slides were incubated with the primary antibody (Supplemental Table 10) followed with Novolink Polymer (Leica Biosystems, RE7260-K) or Vector ImmPRESS (MP-7444-15) secondaries. Antibody detection with DAB was performed using the Bond Intense R detection system (Leica Biosystems, DS9263). Stained slides were dehydrated and coverslipped with Cytoseal 60 (23-244256, Thermo Fisher Scientific). Positive controls were included for each assay. IHC-stained slides were digitally imaged in the Aperio ScanScope XT (Leica Biosystems) using 20× objective.

IHC for CK5 and p63 was performed on 5 μm paraffin sections. Slides were dewaxed in xylene and rehydrated in ethanol before undergoing antigen retrieval (Vector Lab 3300) for 20 minutes. Endogenous peroxidase was quenched using 3% hydrogen peroxide followed by avidin and biotin blocking (Vector Lab SP-2001). For CK-5 staining, slides were blocked with 10% goat serum for 1–2 hours at room temperature before incubation with primary rabbit polyclonal anti–CK-5 antibody (1:500 dilution, Abcam ab52635) in 3% goat serum overnight. Washed slides underwent secondary antibody treatment with biotinylated anti-rabbit IgG (Biocare Medical GR608H) for 30 minutes. p63 slides were blocked with avidin and biotin before blocking with the MOM blocking reagent (Vector Lab BMK-2202) for mouse-on-mouse antibodies. p63 slides were incubated with primary mouse antibodies in MOM diluent (p63 Biocare Medical CM163A 1:100). All slides were then incubated with avidin-biotin complexes for 30 minutes and washed before being developed in DAB solution (Impact DAB from Vector Lab SK-4105) and counterstained in hematoxylin (Sigma-Aldrich GHS316).

Triple immunofluorescence (IF) was performed on paraffin-embedded tissues that were sectioned onto slides. Tissue sections were sequentially labeled for antigens using a triple stain assay with the following antibodies of interest: HNF-4a (Abcam, ab201460), Ki-67 (Cell Signaling Technology, 12202S), TTF1 (Abcam, ab133737), KRT5 (Cell Signaling Technology, 71536), and p63 (Proteintech, 12143-1-AP). This IF assay was carried out on the Bond III fully automated slide staining system (Leica Biosystems) using the Bond Research Detection kit (DS9455). Slides were deparaffinized in Leica Biosystems Bond Dewax solution (AR9222), hydrated in Bond Wash solution (AR9590), and sequentially stained for a triplex stain. Briefly, antigen retrieval was accomplished using Bond-Epitope Retrieval solution 1 pH-6.0 or Bond-Epitope Retrieval solution 2 pH-9.0. After pretreatment, tissues were blocked, and primary antibodies were diluted as follows: HNF-4a at 1:5,000, Ki-67 at 1:400, TTF1 at 1:2,000, KRT5 at 1:500, and p63 at 1:200. Secondary antibodies Novolink Polymer (Leica Biosystems, RE7260-CE) were used followed by TSA Cy5 (Akoya Biosciences, SAT705A001EA), TSA Cy3 (Akoya Biosciences, SAT704A001EA), or Alexa Fluor 488 Tyramide Reagent (Thermo Fisher Scientific, B40953) to visualize the target of interest. Nuclei were stained with Hoechst 33258 (Invitrogen). The stained slides were mounted with ProLong Gold antifade reagent (Thermo Fisher Scientific, P36930). A positive control was included in this run.

Slides were digitalized using the Aperio ScanScope FL (Aperio Technologies Inc). The digital images were captured in each channel by 20× objective (0.468 μm/pixel resolution) using line-scan camera technology (US Patent 6,711,283; Aperio Technologies Inc). The adjacent 1 mm stripes captured across the entire slide were aligned into a contiguous digital image by an image composer. Images were archived in UNC Pathology Services Core’s eSlide Manager database (Leica Biosystems).

### Statistics.

Results for each group were compared using 2-tailed Student’s *t* test (for comparisons of 2 groups) and 2-way ANOVA (for multiple-group comparisons). A *P* value less than 0.05 was deemed statistically significant. All statistical tests for in vitro and in vivo experiments were performed using GraphPad Prism 7 (GraphPad Software, Inc.).

### Study approval.

All animal experiments were approved by the University of North Carolina at Chapel Hill Institutional Animal Care and Use Committee (IACUC), and the care of the animals was in accordance with institutional guidelines (IACUC protocol no. 24-088). All animal experiments were conducted in compliance with the NIH *Guide for the Care and Use of Laboratory Animals* (National Academies Press, 2011) for animal research.

### Data availability.

Supplemental datasets generated and analyzed in the current study can be accessed in Supplemental Methods. The bulk sequencing and single-cell sequencing data have been grouped under a SuperSeries: Gene Expression Omnibus GSE295887. Values for all data points in graphs are reported in the Supporting Data Values file.

## Author contributions

CVP conceived the study. NS, WG, JLM, ATW, EWL, KDFS, LE, and KMW developed methodology. NS, WG, JLM, RSS, HY, VG, and CVP investigated. NS, WG, JLM, ATW, and EWL performed formal data analysis and visualization. CVP acquired funding. HY, GPG, and CVP provided resources. NS and CVP wrote the original draft. NS, KMW, JLM, RSS, WDG, WG, ATW, EWL, KDFS, GC, LE, HY, ACC, YS, KZ, AD, JMG, VG, JJM, GPG, and CVP reviewed and edited the manuscript.

## Supplementary Material

Supplemental data

Supplemental tables 1-10

Supporting data values

## Figures and Tables

**Table 1:**
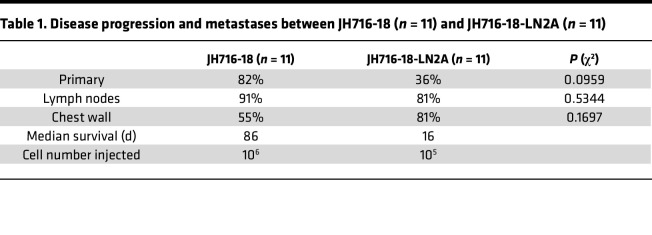
Disease progression and metastases between JH716-18 (*n* = 11) and JH716-18-LN2A (*n* = 11)

## Version 1 05/06/2025

In-Press Preview

## Version 2 06/23/2025

Electronic publication

## References

[1] https://www.cancer.org/content/dam/cancer-org/research/cancer-facts-and-statistics/global-cancer-facts-and-figures/global-cancer-facts-and-figures-2024.pdf.

[2] Siegel RL (2022). Cancer statistics, 2022. CA Cancer J Clin.

[3] Chen Z (2014). Non-small-cell lung cancers: a heterogeneous set of diseases. Nat Rev Cancer.

[4] Hynds RE (2021). et al. Progress towards non-small-cell lung cancer models that represent clinical evolutionary trajectories. Open Biol.

[5] Ferone G (2020). Cells of origin of lung cancers: lessons from mouse studies. Genes Dev.

[6] Inamura K (2018). Update on immunohistochemistry for the diagnosis of lung cancer. Cancers (Basel).

[7] Kris MG (2014). Using multiplexed assays of oncogenic drivers in lung cancers to select targeted drugs. JAMA.

[8] Justilien V (2014). The PRKCI and SOX2 oncogenes are coamplified and cooperate to activate Hedgehog signaling in lung squamous cell carcinoma. Cancer Cell.

[9] Tata PR (2018). Developmental history provides a roadmap for the emergence of tumor plasticity. Dev Cell.

[10] Mukhopadhyay A (2014). Sox2 cooperates with Lkb1 loss in a mouse model of squamous cell lung cancer. Cell Rep.

[11] Mollaoglu G (2018). The lineage-defining transcription factors SOX2 and NKX2-1 determine lung cancer cell fate and shape the tumor immune microenvironment. Immunity.

[12] Cancer Genome Atlas Research Network (2012). Comprehensive genomic characterization of squamous cell lung cancers. Nature.

[13] Cancer Genome Atlas Research Network (2014). Comprehensive molecular profiling of lung adenocarcinoma. Nature.

[14] Jamal-Hanjani M (2017). Tracking the evolution of non-small-cell lung cancer. N Engl J Med.

[15] McFadden DG (2016). Mutational landscape of EGFR-, MYC-, and Kras-driven genetically engineered mouse models of lung adenocarcinoma. Proc Natl Acad Sci U S A.

[16] Lu Y (2010). Evidence that SOX2 overexpression is oncogenic in the lung. PLoS One.

[17] Ferone G (2016). SOX2 is the determining oncogenic switch in promoting lung squamous cell carcinoma from different cells of origin. Cancer Cell.

[18] Hai J (2020). Generation of genetically engineered mouse lung organoid models for squamous cell lung cancers allows for the study of combinatorial immunotherapy. Clin Cancer Res.

[19] Afshar-Kharghan V (2017). The role of the complement system in cancer. J Clin Invest.

[20] Walter DM (2017). Systematic in vivo inactivation of chromatin-regulating enzymes identifies Setd2 as a potent tumor suppressor in lung adenocarcinoma. Cancer Res.

[21] DuPage M (2009). Conditional mouse lung cancer models using adenoviral or lentiviral delivery of Cre recombinase. Nat Protoc.

[22] Harrison EB (2020). A circle RNA regulatory axis promotes lung squamous metastasis via CDR1-mediated regulation of Golgi trafficking. Cancer Res.

[23] Porrello A (2018). Factor XIIIA-expressing inflammatory monocytes promote lung squamous cancer through fibrin cross-linking. Nat Commun.

[24] Castellano GM (2022). Inhibition of Mtorc1/2 and DNA-PK via CC-115 synergizes with carboplatin and paclitaxel in lung squamous cell carcinoma. Mol Cancer Ther.

[25] Alzahrani AS (2019). PI3K/Akt/mTOR inhibitors in cancer: at the bench and bedside. Semin Cancer Biol.

[26] Madden E (2019). The role of the unfolded protein response in cancer progression: from oncogenesis to chemoresistance. Biol Cell.

[27] Kargi A (2007). The diagnostic value of TTF-1, CK 5/6, and p63 immunostaining in classification of lung carcinomas. Appl Immunohistochem Mol Morphol.

[28] Relli V (2018). Distinct lung cancer subtypes associate to distinct drivers of tumor progression. Oncotarget.

[29] Zhang H (2017). Lkb1 inactivation drives lung cancer lineage switching governed by Polycomb Repressive Complex 2. Nat Commun.

[30] Travis WD (2011). International Association for the Study of Lung Cancer/American Thoracic Society/European Respiratory Society international multidisciplinary classification of lung adenocarcinoma. J Thorac Oncol.

[31] Zilionis R (2019). Single-cell transcriptomics of human and mouse lung cancers reveals conserved myeloid populations across individuals and species. Immunity.

[32] Tsui KH (2012). Glycoprotein transmembrane nmb: an androgen-downregulated gene attenuates cell invasion and tumorigenesis in prostate carcinoma cells. Prostate.

[33] Wellenstein MD (2019). p53 loss can promote neutrophilia, increased WNT signaling, and metastasis. Cancer Discov.

[34] Bachem A (2012). Expression of XCR1 characterizes the Batf3-dependent lineage of dendritic cells capable of antigen cross-presentation. Front Immunol.

[35] Hildner K (2008). Batf3 deficiency reveals a critical role for CD8alpha^+^ dendritic cells in cytotoxic T cell immunity. Science.

[36] Lund AW, De Palma M, Targeting LRG1 boosts immunotherapy (2021). Med.

[37] O’Connor MN (2021). LRG1 destabilizes tumor vessels and restricts immunotherapeutic potency. Med.

[38] Zhong B (2021). Colorectal cancer-associated fibroblasts promote metastasis by up-regulating LRG1 through stromal IL-6/STAT3 signaling. Cell Death Dis.

[39] Cai D (2022). LRG1 in pancreatic cancer cells promotes inflammatory factor synthesis and the angiogenesis of HUVECs by activating VEGFR signaling. J Gastrointest Oncol.

[40] Choi CHJ (2022). LRG1 is an adipokine that promotes insulin sensitivity and suppresses inflammation. Elife.

[41] Neumann D (2000). The membrane form of the type II IL-1 receptor accounts for inhibitory function. J Immunol.

[42] Pou J (2011). Type II interleukin-1 receptor expression is reduced in monocytes/macrophages and atherosclerotic lesions. Biochim Biophys Acta.

[43] Karakasheva TA (2015). CD38-expressing myeloid-derived suppressor cells promote tumor growth in a murine model of esophageal cancer. Cancer Res.

[44] Karakasheva TA (2018). CD38^+^ M-MDSC expansion characterizes a subset of advanced colorectal cancer patients. JCI Insight.

[45] Wu F (2021). Single-cell profiling of tumor heterogeneity and the microenvironment in advanced non-small cell lung cancer. Nat Commun.

[46] He C (2021). Low CFB expression is independently associated with poor overall and disease‑free survival in patients with lung adenocarcinoma. Oncol Lett.

[47] Wu P (2021). The prognostic value of plasma complement factor B (CFB) in thyroid carcinoma. Bioengineered.

[48] Pozzi S (2022). MCP-1/CCR2 axis inhibition sensitizes the brain microenvironment against melanoma brain metastasis progression. JCI Insight.

[49] Serra P (2018). Programmed cell death-ligand 1 (PD-L1) expression is associated with RAS/TP53 mutations in lung adenocarcinoma. Lung Cancer.

[50] Jackson EL (2005). The differential effects of mutant p53 alleles on advanced murine lung cancer. Cancer Res.

[51] Gkountakos A (2019). PTEN in lung cancer: dealing with the problem, building on new knowledge and turning the game around. Cancers (Basel).

[52] Lane DP (1992). Cancer. p53, guardian of the genome. Nature.

[53] Verduci L (2017). The oncogenic role of circPVT1 in head and neck squamous cell carcinoma is mediated through the mutant p53/YAP/TEAD transcription-competent complex. Genome Biol.

[54] Wang W (2013). Mutant p53-R273H gains new function in sustained activation of EGFR signaling via suppressing miR-27a expression. Cell Death Dis.

[55] Zhu J (2015). Gain-of-function p53 mutants co-opt chromatin pathways to drive cancer growth. Nature.

[56] Eriksson M (2017). Effect of mutant p53 proteins on glycolysis and mitochondrial metabolism. Mol Cell Biol.

[57] Liu J (2019). Tumor suppressor p53 and metabolism. J Mol Cell Biol.

[58] Dong P (2013). Mutant p53 gain-of-function induces epithelial-mesenchymal transition through modulation of the miR-130b-ZEB1 axis. Oncogene.

[59] Wang SP (2009). p53 controls cancer cell invasion by inducing the MDM2-mediated degradation of Slug. Nat Cell Biol.

[60] Kogan-Sakin I (2011). Mutant p53(R175H) upregulates Twist1 expression and promotes epithelial-mesenchymal transition in immortalized prostate cells. Cell Death Differ.

[61] Lu C, El-Deiry WS (2009). Targeting p53 for enhanced radio- and chemo-sensitivity. Apoptosis.

[62] Zhu G (2020). Mutant p53 in cancer progression and targeted therapies. Front Oncol.

[63] Stein Y (2019). Mutant p53-a potential player in shaping the tumor-stroma crosstalk. J Mol Cell Biol.

[64] Cordani M (2016). Mutant p53 proteins alter cancer cell secretome and tumour microenvironment: involvement in cancer invasion and metastasis. Cancer Lett.

[65] Loging WT, Reisman D (1999). Inhibition of the putative tumor suppressor gene TIMP-3 by tumor-derived p53 mutants and wild type p53. Oncogene.

[66] Rahnamoun H (2017). Mutant p53 shapes the enhancer landscape of cancer cells in response to chronic immune signaling. Nat Commun.

[67] Hou Y (2021). Significance of *TP53* mutation in cellular process and disease progression in lung adenocarcinoma. Genet Test Mol Biomarkers.

[68] Keniry M, Parsons R (2008). The role of PTEN signaling perturbations in cancer and in targeted therapy. Oncogene.

[69] Ji H (2007). LKB1 modulates lung cancer differentiation and metastasis. Nature.

[70] Han X (2014). Transdifferentiation of lung adenocarcinoma in mice with Lkb1 deficiency to squamous cell carcinoma. Nat Commun.

[71] Crystal RG (2008). Airway epithelial cells: current concepts and challenges. Proc Am Thorac Soc.

[72] Xie GD (2018). Epidemiology and survival outcomes of mucinous adenocarcinomas: a SEER population-based study. Sci Rep.

[73] Snyder EL (2013). Nkx2-1 represses a latent gastric differentiation program in lung adenocarcinoma. Mol Cell.

[74] Cantley LC, Neel BG (1999). New insights into tumor suppression: PTEN suppresses tumor formation by restraining the phosphoinositide 3-kinase/AKT pathway. Proc Natl Acad Sci U S A.

[75] Yuan TL, Cantley LC (2008). PI3K pathway alterations in cancer: variations on a theme. Oncogene.

[76] Zhang L (2020). Identification of the key genes and characterizations of Tumor Immune Microenvironment in lung adenocarcinoma (LUAD) and lung squamous cell carcinoma (LUSC). J Cancer.

[77] Chen L (2018). CD38-mediated immunosuppression as a mechanism of tumor cell escape from PD-1/PD-L1 blockade. Cancer Discov.

[78] Pillai RN (2020). Daratumumab plus atezolizumab in previously treated advanced or metastatic NSCLC: brief report on a randomized, open-label, phase 1b/2 study (LUC2001 JNJ-54767414). JTO Clin Res Rep.

[79] Xue W (2014). CRISPR-mediated direct mutation of cancer genes in the mouse liver. Nature.

[80] Aubrey BJ (2015). An inducible lentiviral guide RNA platform enables the identification of tumor-essential genes and tumor-promoting mutations in vivo. Cell Rep.

[81] Sánchez-Rivera FJ (2014). Rapid modelling of cooperating genetic events in cancer through somatic genome editing. Nature.

[82] Matsumoto M (1997). Abrogation of the alternative complement pathway by targeted deletion of murine factor B. Proc Natl Acad Sci U S A.

