# Supplementary material for: Tumor suppressors in Sox2-mediated lung cancers promote distinct cell-intrinsic and immunologic remodeling
Source: JCI Insight. 2025 May 6;10(12):e171364. doi: 10.1172/jci.insight.171364 (PMC12220946; doi:10.1172/jci.insight.171364)
Supplement: Supplemental data [file jciinsight-10-171364-s007.pdf]

Tumor suppressors in Sox2-mediated lung cancers promote distinct cell intrinsic and immunologic remodeling

Authors: Nisitha Sengottuvel*, Kristina M. Whately*, Jennifer L. Modliszewski*, Rani S. Sellers, William D. Green, Weida Gong, Allison T. Woods, Eric W. Livingston, Katerina D. Fagan-Solis, Gabrielle Cannon, Lincy Edatt, Hong Yuan, Aaron C. Chack, Yazmin Sanchez, Katherine Zhou, Alyaa Dawoud, Jarred M. Green, Virginia Godfrey, J Justin Milner, Gaorav P. Gupta, Chad V. Pecot

Supplemental Methods

Flow Cytometry

Single-cell suspensions of tumors were incubated with 1:800 FcR blocking antibody (BioLegend) and subsequently stained with LIVE/DEAD Fixable Blue (Invitrogen) for 10min at room temperature. Flow cytometry antibody master mixes were prepared in FACS buffer containing 2% FBS in PBS. Cells were stained for 30min. For intracellular staining we used the Foxp3/Transcription Factor Staining Buffer Set (Invitrogen) and stained for 45mins at 4°C. Samples were analyzed on a Cytek Aurora 5L spectral flow cytometer. FlowJo v10 (BD) was used for gating and analysis. We used the following antibodies: CD45 BUV395 (BD, #564279, Clone: 30-F11), CD44 BUV536 (BD, #741227, Clone: IM7), CD80 BUV 661 (BD, #741515, Clone: 16-10A1), CD11c BUV737 (BD, #564986, Clone: HL3), HLA-A/B/C BV421 (BD, #565332, Clone: G46-2.6), CD38 eFluor450 (eBioscience, #48-0381-82, Clone: 90), Ly6G BV510 (BioLegend, #127633, Clone: 1A8), CD62L BV570 (BioLegend, #104433, Clone: MEL-14), CD115 BV605 (BioLegend, # 135517, Clone: AFS98), CXCR5 BV650 (BioLegend, # 145517, Clone: L138D7), CD138 BV750 (BD, # 747070, Clone: 281-2), F4/80 BV785 (BioLegend, # 123141, Clone: MB8), IA-IE Spark Blue 550 (BioLegend, # 107661, Clone: M114.1), CD11b PerCP-Cy5.5 (BioLegend, #101228, Clone: M1/70), CD8a Alexa Fluor 594 (BioLegend, #100758, Clone: 53-6.7), CD19 PE/Cy5.5 (Thermo Fisher, 35-0193-82, Clone: eBio1D3), CD103 PE-Cy7 (BioLegend, #121426, Clone: 2 E7), B220 PE-Fire810 (BioLegend, #103287, Clone: RA3-6B2), SiglecF Alexa Fluor 647 (BD, #562680, Clone: E50-2440), CD3 Alexa Fluor 700 (BioLegend, #100216, Clone: 17A2), Ly6C APC/Fire810 (BioLegend, #128055, Clone: HK1.4), and Live/Dead Fixable Blue (Invitrogen, L34961).

Computed Tomography (CT) Scans and Analysis

In vivo animal CT imaging was conducted at the UNC Small Animal Imaging Core facility. A high-resolution animal CT system (Quantum GX2, PerkinElmer Inc, Waltham MA) was used to carry

out retrospective respiration-gated CT imaging of mouse lungs. Animals were anesthetized using isoflurane (3% for induction and 2% for maintenance) mixed with oxygen throughout the imaging procedure. Raw X-ray projections were acquired for 4 min with the following parameters: 90 kV peak voltage, 88 µA current, 40mm FOV in Z direction, and high-speed acquisition mode. Gating parameters were derived based on diaphragm movement in raw projection images and used to generate two sets of projections in two phases, inspiration and expiration phase. Binned projections were reconstructed using Feldkamp algorithm to form lung CT images in inspiration and expiration phases with final voxel size of 120 micron. Radiation dose from each acquisition was approximately 0.2 Gy on X-ray exposed tissue. CT images were viewed using the ITK-SNAP program (Version 3.8.0) and annotated for tumor diameter length. Mice were monitored monthly and once a tumor reached 5mm diameter in any one direction, the mouse was harvested for tissue collection.

Dissociation and Cryopreservation of Tumors

Mice were sacrificed via cervical dislocation and then tumors were dissected from the lung and placed in cold DMEM + Y27632 (ROCK Inhibitor, Selleckchem #S1049) (1:1000) and DNAse I (Worthington Biochemical Corporation #LS002058) (1:50) until ready for dissociation. Tumor tissue was cut into small pieces then dissociated in a solution of HBSS, Y27632 (1:1000), DNase I (1:50) and Protease from *Bacillus licheniformis* (Sigma #P5380) (1:20). The tumor suspension was triturated through a cut pipette tip then placed on a rocker in a cold room. This process was repeated every five minutes until the tumor was visibly broken down. Cells were filtered through a 70 µM filter and washed with fully supplemented DMEM. Cells were pelleted and cryopreserved with FBS + Y27632 (1:500), DNase I (1:50) and DMSO (1:10).

Single Cell Library Prep and Single Cell Sequencing

Dissociated tumor cell viability of each sample was checked by acridine orange and propidium iodide staining using the LUNA-FL Dual Fluorescence Cell Counter (Logos Biosystems) and concentrated to 800 live cells/μL in 0.04% BSA in PBS. The 10X Genomics Chromium Controller and the Chromium Single Cell 3' GEM, Library & Gel Bead Kit v3 (PN-100075) were used for processing cells according to the manufacturer's recommendations (Document#CG000183 Rev C). Library preparation was done by loading approximately 16,000 cells per sample onto the Chromium Chip B, targeting a 10,000-cell recovery per sample. Gel beads in emulsion (GEMs) were created by encapsulating single cells, reverse transcription reagents and gel beads coated with barcoded oligos in an oil droplet. Reverse transcription was used to create cDNA libraries tagged with a cell barcode and unique molecular index (UMI). cDNA libraries were purified using Dynabeads MyOne SILANE (Invitrogen) before 11 amplification cycles after GEMs were broken. Amplified cDNA libraries were purified with SPRIselect magnetic beads (Beckman Coulter) and quantified using an Agilent Bioanalyzer High Sensitivity DNA chip (Agilent Technologies). After these SPRIselect beads were used for fragmentation, end repair, A-tailing and double-sided size selection, Illumina-compatible adapters were ligated onto the size selected cDNA fragments. These were then purified and amplified with uniquely identifiable indexes. After purification, the finalized sequencing libraries were visualized using the Bioanalyzer High Sensitivity DNA chip, normalized to 20 nM, and pooled.

Pooled libraries were sequenced using a NovaSeq6000S4 machine (Illumina). Libraries were denatured and diluted following standard Illumina protocol, spiked with 1% PhiX sequencing control (Illumina), loaded onto the flow cell and sequenced in paired-end format with 1D indexing. The libraries were read at 28, 8, 16 and 91 cycles for Read 1, i7 index, i5 index and Read 2, respectively. This yielded a depth of 31-141 x 10^6 reads per sample (**Supplementary Table S7**).

Single-cell RNA-Seq analysis

The fastq files were de-multiplexed, aligned, and quantified with 10X Genomics Cell Ranger v3.1.0. GFP sequence was added to mm10 reference transcriptome to build a custom transcriptome reference. Reads were aligned to the reference and UMI count matrices were obtained for downstream analyses with Seurat v4.0.3 (1). Cells were then filtered in the following steps: 1) cells with >5% mitochondrial genes and < 200 expressed genes were filtered; 2) cells with high nCount_RNA and nFeature_RNA was detected and filtered using 'isOutlier' function in scuttle v1.0.4 (2); 3) doublet cells were detected and filtered with scDblFinder v1.4.0 (3). Data normalization was performed via SCTransform (4) and integration via CCA. Dimension reduction, clustering and uniform manifold approximation and projection (UMAP) were performed using Seurat with default parameters (5). Cell type annotation was performed using a curated list of cell type markers modified from Zilionis et al. 2019 (**Supplementary Table S8**), as well as references to the cluster assignments and SingleR ImmGen annotation (**Supplementary Figure 6 and 7**) (6-8). The immune cells were annotated based on marker gene list from Zilionis et al. For each immune cell type (e.g., neutrophil), we computed the mean of expression of marker genes in each corresponding immune subtype (e.g., N1, N2) and the immune subtype with the highest score was assigned to the cell.

Subclustering

Mac1 cells were normalized by variance stabilizing transformation via SCTransform (4) (v0.3.3) in Seurat (1) (v4.1.1) utilizing 2000 variable features. Integration of Mac1 cells was performed on the sample level utilizing Harmony (9) (v1.0). Mono1 cells were log normalized and scaled; and merged without integration due to sample size. Clustering of cells was performed with the Louvain algorithm (10) with multi-level refinements at multiple resolution after dimensional reduction via PCA and UMAP. Differentially expressed genes among clusters (cluster markers) were identified via a Wilcoxon Rank Sum Test. Genes were included in the cluster marker analysis if they had a log fold change of 0.5 and were found in at least 30% of the cells in the cluster of interest.

Differential Expression Analysis

Pseudobulk matrices were constructed with scater v1.18.6 for differential expression analysis and gene set variation analysis (GSVA). Briefly, cells of the same type were aggregated across biological replicates to be transformed into a gene-sample matrix and filtered to exclude genes with low overall expression at the pseudo-bulk level. Differential expression analysis was performed using likelihood ratio test with edgeR v3.32.1 comparing LN2A, SNL-Trp53, SNL-Pten to SNL-SH (11-13) . Pathway analyses were performed on C2: curated gene sets in the molecular signature database (14). Differentially expressed genes were analyzed with the use of QIAGEN IPA (QIAGEN Inc., https://digitalinsights.qiagen.com/IPA).

Gene Set Analysis

The curated pathway gene sets for mouse (M2, the mouse equivalent of human C2 gene sets) were obtained from MSigDB (v2022.1) (15). The mouse genes were provided as converted gene symbols, created via Ensembl BioMart. Enrichment scores were calculated on the pseudo-bulk level on variance-stabilized gene counts utilizing the “gsva” method from the GSVA Bioconductor package (16) (v.1.42.0). A linear model was implemented to test for differences in gene set enrichment scores via limma (17) (v3.50.3).

Signature Scoring

We curated a marker set for adenocarcinoma-like versus squamous tumor cells. For the SNL Adeno vs. Squamous differential expression analysis, we assigned subtype to a tumor cell by computing the mean of expression for each of the two signatures for each cell and the subtype with the highest score was assigned to the cell. UCell was used to calculate adenocarcinoma scores, squamous scores, mucin scores, and proliferation scores with signatures provided in **Supplementary Tables S1 and S9** (18, 19).

Human Dataset Analysis

Raw counts from human single-cell RNA-Seq samples from LUSC patients published in Wu et al.,(20) were downloaded from GEO (GSE148071). No additional filtering was performed on the dataset, as it appeared to have been filtered prior to upload. The data was pre-processed prior to clustering following default parameters in Seurat (v5.1.0) (4). In short, RNA raw counts were log-normalized, variable features were identified, followed by scaling and PCA. Identification of neighbors and clusters was performed on the first 30 principal components. Clustering was performed via the modified Louvain algorithm at a resolution of 0.5, and UMAP was utilized for further dimensional reduction; and was performed on the first 30 principal components. Clustering and UMAP dimensional reduction were also performed on a Harmony (v1.2.0) (9) integrated data set.

The data were subset to myeloid cells by clustering and examination of marker genes. Following annotation with the minor subset from the human Zilionis lung data via SingleR (v2.0.0), clusters containing dendritic cells, MacCycl, and ambiguous cells were removed, as well as all cells from non-specific NSCLC patients. Signature scores for inflammatory monocytes (Porello, Sengottuvel) and MDSC (Porelllo et al.) were calculated via UCell. Linear mixed effects models with cancer class (LUSC vs LUAD) as a fixed effect and patient ID as a random effect were used to test for differences in inflammatory signatures by cancer class.

Bulk RNA Seq

RNA was isolated from JH716-18, JH716-18-LN1A, and JH716-18-LN2A cell lines using the Quick-RNA MicroPrep kit (Zymo Research, R1051). Purified RNA was quantified using QuBit RNA BR Assay (Thermo Fisher, Q10210). Libraries were made using Illumina TruSeq Stranded mRNA kit and ran on the NextSeq2000 machine generating paired-end 50bp reads. Libraries and sequencing were done by the UNC Translational Genomics Lab. Raw reads were aligned to GRCm38.p6 mouse genome annotated with the GENCODE vM transcriptome via STAR (v2.7.6.a) (21, 22). Transcripts were counted with Salmon (v1.4.0) (23). QC metrics from Picard

CollectRnaSeqMetrics (v2.22.4) and FastQC (v0.11.9) were aggregated with MultiQC (v1.11) (24-26). Raw counts were normalized via DESeq (v.138.0) after removing genes that did not have at least 10 counts in one sample (27). Assessment of QC metrics PCA was performed on data to ensure sample quality. Differential expression analysis was conducted in DESeq2, and a Wald test was utilized to test for differences in groups. Independent filtering was not conducted and p-values were not altered via Cook's cutoff. Gene set enrichment analysis (GSEA) was performed via fgsea (v1.24.0) utilizing the MSgiDB mouse Hallmark (MH), Curated Pathways (M2), positional (M1), and GO biological processes (M5 GO BP) gene sets (v2023.2) (14, 15, 28). Minimum gene set size was set to 15 and maximum gene set size was set to 500.

TCGA correlation analysis

STAR counts from primary tumor samples were downloaded from the NCI GDC data portal[16] for all TCGA projects with available primary tumor tissue samples; counts were aligned to version hg38 of the human genome and Gencode v36 of the human transcriptome. Sample metadata was downloaded using GDC API calls (29). Raw counts for all samples were normalized in DESeq2 (30), after excluding genes that did not have at least 10 counts in at least 10% of the samples. Spearman's rho was calculated to assess correlation between *CFB, PD1 (PDCD1) and PD-L1 (CD274).*

HDL Assembly, Mucinous and inflammatory signatures in TCGA lung cancer

TCGA LUAD and LUSC patients with alterations in *P53* and *PTEN* were identified via cBioPortal (31). Primary tumor samples were utilized from all patients with information about *P53* and *PTEN* mutation and copy number. The alterations data was downloaded from the "Type of Genetic Alterations Across All Samples" table in the downloads tab of cBioPortal after querying for TCGA-LUSC and TCGA-LUAD with aberrations in p53 and PTEN. Raw counts for all samples were normalized in DESeq2 (30) (v1.38.0), after excluding genes that did not have at least 10 counts

in at least 10% of the samples. The final dataset included 970 samples. GSVA (16) (v) was utilized to calculate gene set scores on the individual sample level for mucinosity and the Reactome HDL Assembly gene set from the C2 pathways (MSigDB v2023.1) (32). A Wilcoxon rank-sum test was used to test for differences in HDL assembly and mucinosity signatures by p53 and PTEN status in each dataset independently.

Inflammatory monocyte signature in LUSC by SOX2 status

The signatures for myeloid-derived suppressor cells and inflammatory monocytes identified in Porrello et al. (33) was utilized to assess differences in *SOX2* low and *SOX2*+ samples based on *P53* and *PTEN* in TCGA-LUSC samples. Samples were categorized as *SOX2* low if they had an expression value below the first quartile and *SOX2*+ if they have an expression value at or above the first quartile. Wilcoxon rank-sum tests were used to test for differences across *P53* and *PTEN* alteration status.

SOX2 survival analysis

Clinical data for TCGA-LUSC samples was downloaded from cBioPortal (31). Kruskal-Wallis and Wilcoxon rank-sum tests were used to test for differences in *SOX2* expression across tumor stage. Samples were split into *SOX2* high and *SOX2* low groups based on median *SOX2* expression. Kaplan-Meier survival curves for were estimated with the survival package (34) (v3.6-4) and plotted via survminer (35) (0.4.9), utilizing both overall survival and disease-free survival. Log rank tests were utilized to test for differences in survival curves between the two groups.

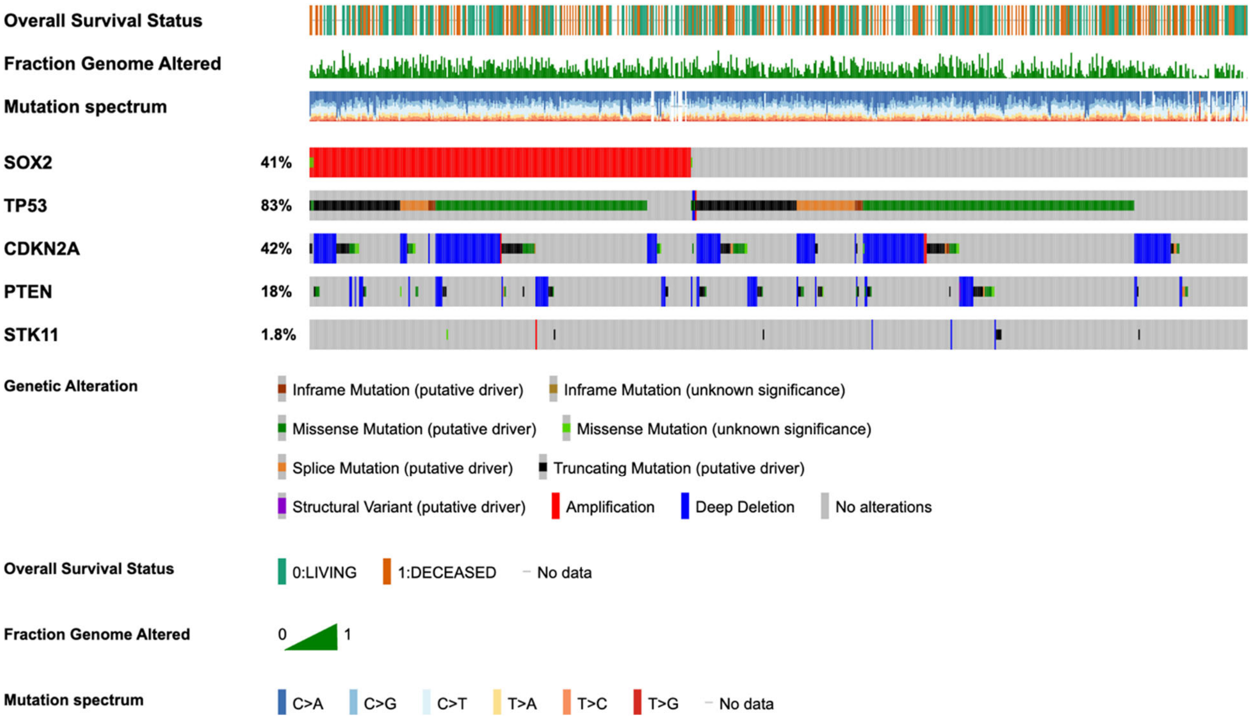

Supplementary Figure 1: SOX2 Copy Number Status and Common mutations in LUSC. cBioportal epidemiological data on mutation burden and CAN data from the Lung Squamous Cell Carcinoma TCGA PanCancer Atlas on cBioportal. Samples were queried by gene mutations for *SOX2*, *TP53*, *CDKN2A, PTEN* and *STK11/LKB1* on overall survival, fraction genome altered, and mutation spectrum.

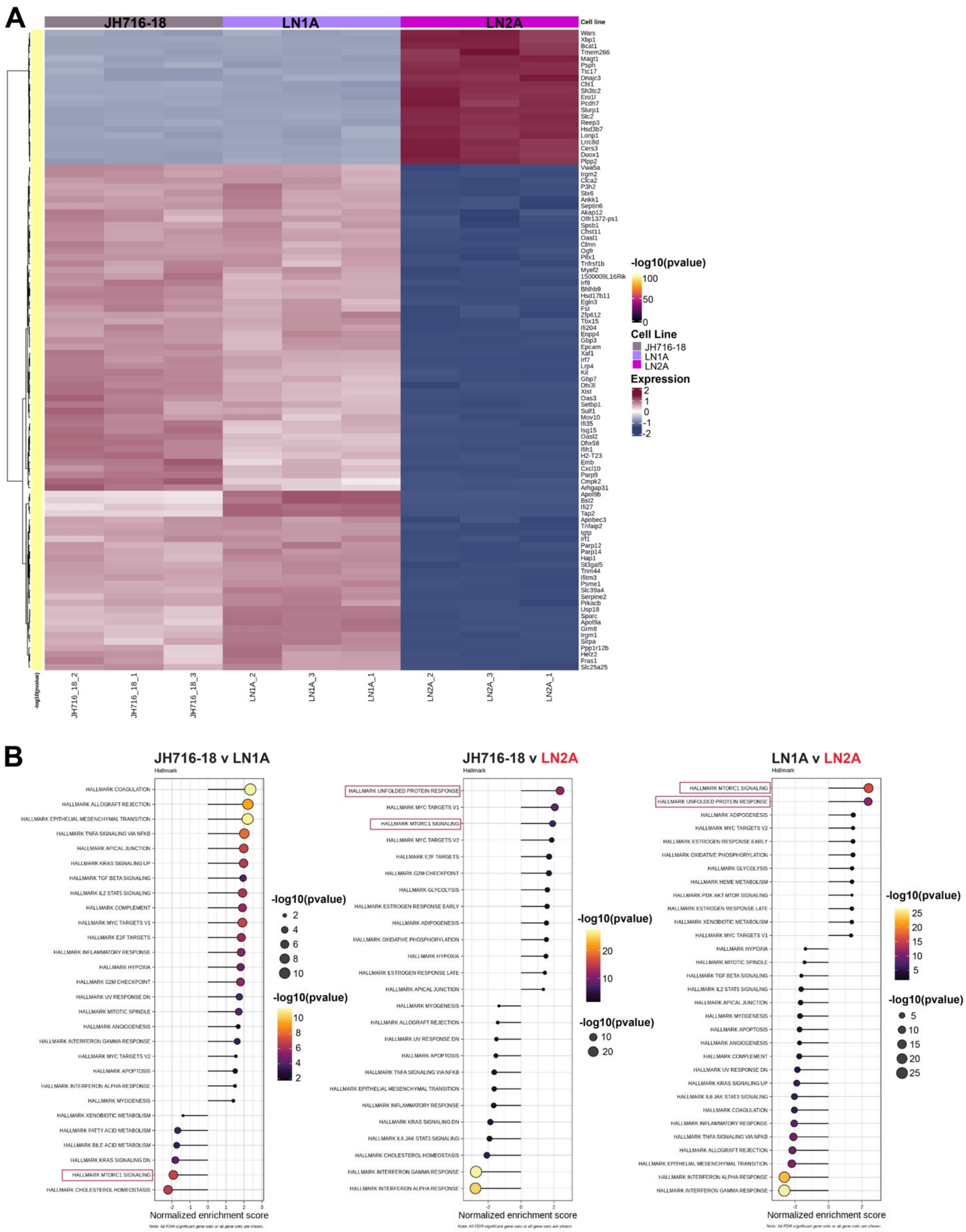
A
JH716-18
LN1A
LN2A
Cell line
Wars
Xbp1
Bcat1
Tmem266
Magt1
Psph
Ttc17
Dnajc3
Chl1
Sh3tc2
Ero1l
Pcdh7
Slurp1
Stc2
Reep3
Hsd3b7
Lonp1
Lrrc8d
Cers3
Duox1
Plpp2
Vwa5a
Irgm2
Clca2
P3h2
Stx6
Ankk1
Septin6
Akap12
Olfr1372-ps1
Spsb1
Chst11
Oasl1
Clmn
Ogfr
Pitx1
Tnfrsf1b
Myef2
1500009L16Rik
Irf9
Bhlhb9
Hsd17b11
Egln3
Fst
Zfp612
Tbx15
Ifi204
Enpp4
Gbp3
Epcam
Xaf1
Irf7
Lrp4
Kit
Gbp7
Dtx3l
Xist
Oas3
Setbp1
Sulf1
Mov10
Ifi35
Isg15
Oasl2
Dhx58
Ifih1
H2-T23
Emb
Cxcl10
Parp9
Cmpk2
Arhgap31
Apol9b
Bst2
Ifi27
Tap2
Apobec3
Tnfaip2
Igtp
Irf1
Parp12
Parp14
Hap1
St3gal5
Trim44
Ifitm3
Psme1
Slc39a4
Serpine2
Prkacb
Usp18
Sparc
Apol9a
Grm8
Irgm1
Sirpa
Ppp1r12b
Helz2
Fras1
Slc25a25
-log10(pvalue)
JH716_18_2
JH716_18_1
JH716_18_3
LN1A_2
LN1A_3
LN1A_1
LN2A_2
LN2A_3
LN2A_1
-log10(pvalue)
100
50
0
Cell Line
JH716-18
LN1A
LN2A
Expression
2
1
0
-1
-2
B
JH716-18 v LN1A
Hallmark
HALLMARK COAGULATION
HALLMARK ALLOGRAFT REJECTION
HALLMARK EPITHELIAL MESENCHYMAL TRANSITION
HALLMARK TNFA SIGNALING VIA NFKB
HALLMARK APICAL JUNCTION
HALLMARK KRAS SIGNALING UP
HALLMARK TGF BETA SIGNALING
HALLMARK IL2 STAT5 SIGNALING
HALLMARK COMPLEMENT
HALLMARK MYC TARGETS V1
HALLMARK E2F TARGETS
HALLMARK INFLAMMATORY RESPONSE
HALLMARK HYPOXIA
HALLMARK G2M CHECKPOINT
HALLMARK UV RESPONSE DN
HALLMARK MITOTIC SPINDLE
HALLMARK ANGIOGENESIS
HALLMARK INTERFERON GAMMA RESPONSE
HALLMARK MYC TARGETS V2
HALLMARK APOPTOSIS
HALLMARK INTERFERON ALPHA RESPONSE
HALLMARK MYOGENESIS
HALLMARK XENOBIOTIC METABOLISM
HALLMARK FATTY ACID METABOLISM
HALLMARK BILE ACID METABOLISM
HALLMARK KRAS SIGNALING DN
HALLMARK MTORC1 SIGNALING
HALLMARK CHOLESTEROL HOMEOSTASIS
-2 -1 0 1 2 3
Normalized enrichment score
-log10(pvalue)
2
4
6
8
10
-log10(pvalue)
10
8
6
4
2
JH716-18 v LN2A
Hallmark
HALLMARK UNFOLDED PROTEIN RESPONSE
HALLMARK MYC TARGETS V1
HALLMARK MTORC1 SIGNALING
HALLMARK MYC TARGETS V2
HALLMARK E2F TARGETS
HALLMARK G2M CHECKPOINT
HALLMARK GLYCOLYSIS
HALLMARK ESTROGEN RESPONSE EARLY
HALLMARK ADIPOGENESIS
HALLMARK OXIDATIVE PHOSPHORYLATION
HALLMARK HYPOXIA
HALLMARK ESTROGEN RESPONSE LATE
HALLMARK APICAL JUNCTION
HALLMARK MYOGENESIS
HALLMARK ALLOGRAFT REJECTION
HALLMARK UV RESPONSE DN
HALLMARK APOPTOSIS
HALLMARK TNFA SIGNALING VIA NFKB
HALLMARK EPITHELIAL MESENCHYMAL TRANSITION
HALLMARK INFLAMMATORY RESPONSE
HALLMARK KRAS SIGNALING DN
HALLMARK IL6 JAK STAT3 SIGNALING
HALLMARK CHOLESTEROL HOMEOSTASIS
HALLMARK INTERFERON GAMMA RESPONSE
HALLMARK INTERFERON ALPHA RESPONSE
-2 0 2
Normalized enrichment score
-log10(pvalue)
20
10
-log10(pvalue)
10
20
LN1A v LN2A
Hallmark
HALLMARK MTORC1 SIGNALING
HALLMARK UNFOLDED PROTEIN RESPONSE
HALLMARK ADIPOGENESIS
HALLMARK MYC TARGETS V2
HALLMARK ESTROGEN RESPONSE EARLY
HALLMARK OXIDATIVE PHOSPHORYLATION
HALLMARK GLYCOLYSIS
HALLMARK HEME METABOLISM
HALLMARK PI3K AKT MTOR SIGNALING
HALLMARK ESTROGEN RESPONSE LATE
HALLMARK XENOBIOTIC METABOLISM
HALLMARK MYC TARGETS V1
HALLMARK HYPOXIA
HALLMARK MITOTIC SPINDLE
HALLMARK TGF BETA SIGNALING
HALLMARK IL2 STAT5 SIGNALING
HALLMARK APICAL JUNCTION
HALLMARK MYOGENESIS
HALLMARK APOPTOSIS
HALLMARK ANGIOGENESIS
HALLMARK COMPLEMENT
HALLMARK UV RESPONSE DN
HALLMARK KRAS SIGNALING UP
HALLMARK IL6 JAK STAT3 SIGNALING
HALLMARK COAGULATION
HALLMARK INFLAMMATORY RESPONSE
HALLMARK TNFA SIGNALING VIA NFKB
HALLMARK ALLOGRAFT REJECTION
HALLMARK EPITHELIAL MESENCHYMAL TRANSITION
HALLMARK INTERFERON ALPHA RESPONSE
HALLMARK INTERFERON GAMMA RESPONSE
-2 0 2
Normalized enrichment score
-log10(pvalue)
25
20
15
10
5
-log10(pvalue)
5
10
15
20
25

Supplementary Figure 2: Bulk RNA-seq results for parental cell lines of LN2A. A) Heatmap displaying the differential expression of genes across three cell lines: JH716-18, LN1A, and LN2A. RNA-seq data were normalized using DESeq2 after filtering genes with fewer than 10 counts in at least one sample. Gene expression values are shown as scaled expression (z-scores), with red indicating higher expression and blue indicating lower expression. Rows represent individual genes, while columns represent biological replicates. Gene list from the top down: *Wars, Ybx3, Gbe1, Nat6, Mgat3, Tlr2, Phpt1, Dnase2, Slc7a2, Chchd2, Pcbp1, Surf1, Ppib, Hspa9, Hsd17b7, Lrrc57, Uros, Pip4k2a, Dnaaf3, Napepld, Gm2a, Psat1, Fshb, Aars, Asns, Sdha, Gfm1, Pigt, Hyprt, 1500009L16Rik, Ndufa9, Eif4g2, Bsph1, Gm5818, Fst, Fis1, Tcf7l2, Tbx5, Eif4ebp4, Eps8, Gdpd1, Kmt2a, Iqcf4, Lrrfip1, Olr1, Xbp1, Osbpl3, Mrps21, Myl9, Myo10, Dhx36, Hsph1, Hmgb1, Tfrc, Ighg, Ap1s3, Ptpn12, Tmem184b, Stip1, Trim14, Itpr2, Tmem4, 5830418K08Rik, Srpk2, Eif4a2, Aarsd1, Igfbp1, Gimap4, Kifap3, Abcc12, Saa3, Tmem72, Prpsap2, Hba-a2, Plekha3, S100a4, Slc25a25.* B) Gene set enrichment analysis (GSEA) results comparing JH716-18 vs. LN1A, JH716-18 vs. LN2A, and LN1A vs. LN2A. Enrichment scores were calculated using the fgsea package with the MSigDB Hallmark gene sets. Dot plots depict normalized enrichment scores (NES) for significantly enriched pathways, with dot size representing -log10(p-value) and color intensity reflecting significance. Key pathways highlighted.

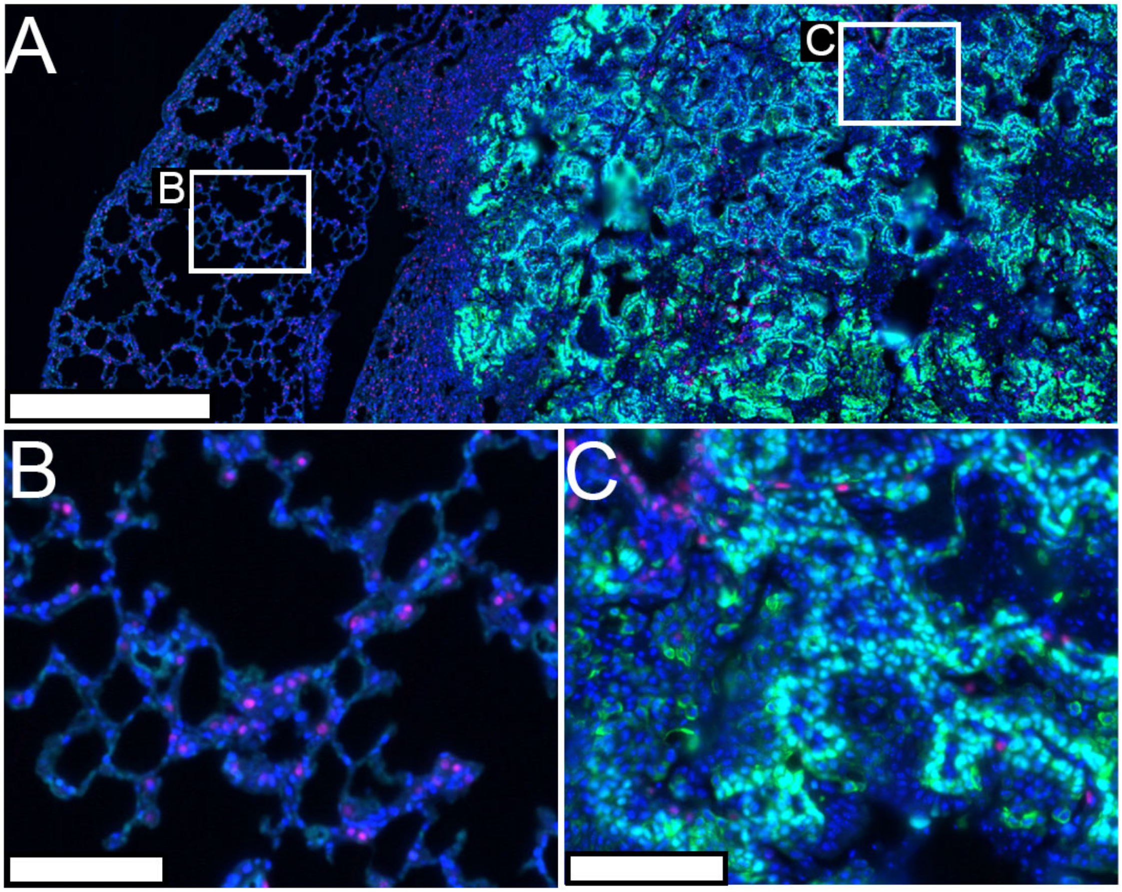

Supplementary Figure 3: Alveolar and tumor staining of TTF1. A) Normal lung and tumor section staining of TTF1 (red) in alveoli (B, inset) surrounding tumoral tissue from the orthotopic LN2A model. LN2A tumor (C, inset) is labeled by the presence of p63 (cyan) and KRT5 (green). Scale Bar = 600 μM (A), Scale Bar = 100 μM (B and C).

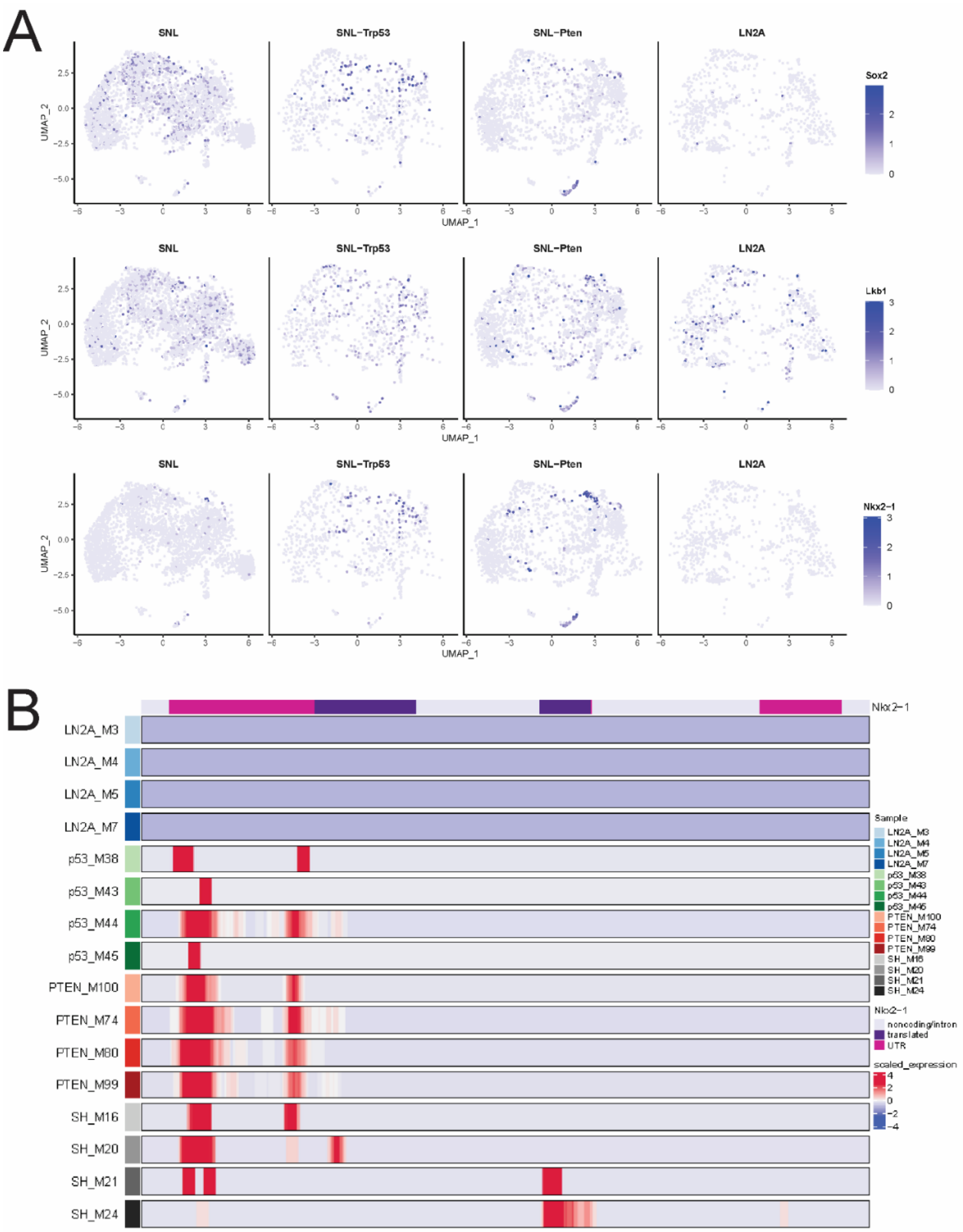

Supplementary Figure 4: Sox2, Lkb1, Nkx2-1 Expression. A) UMAPs of tumor cells (n=6,808 cells) by model, showing the expression of *Sox2*, *Lkb1* (*Stk11*), and *Nkx2-1*. B) Heatmap of scaled depth of read coverage across *Nkx2-1*, by sample, for all tumor cells. Each base pair position of the Nkx2-1-201 transcription +/- 200bp upstream and downstream is a column in the heatmap. The depth of coverage is scaled by sample (row). Only cell-associated reads (xf:i:25 BAM tag) are shown. The gene is transcribed from the negative strand. Base pairs corresponding to exons are annotated as either translated sequence (purple) or UTR (pink). All other positions are either intronic or upstream/downstream.

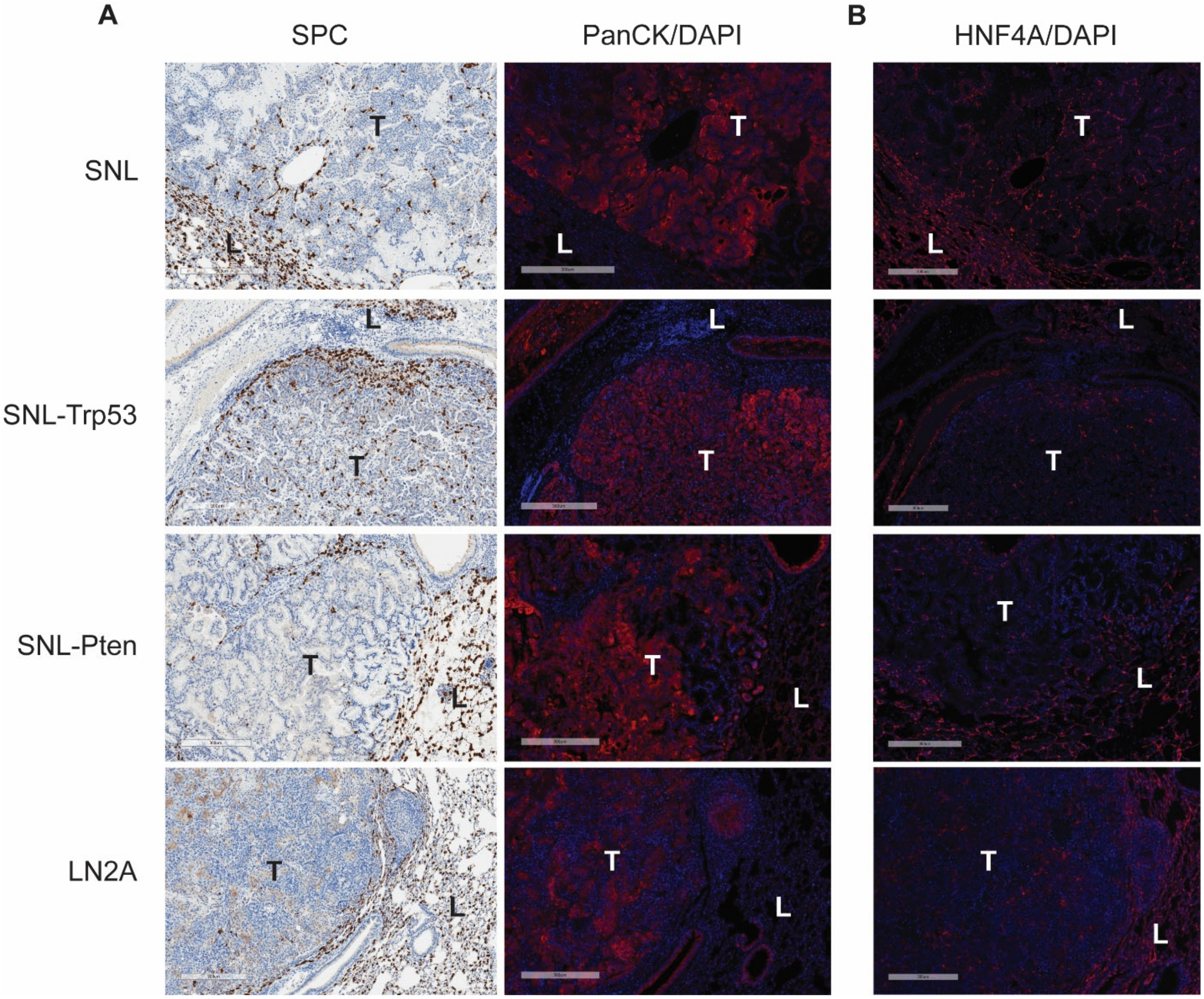

Supplementary Figure 5: Differential SPC, PanCK, and HNF4A Expression in Mouse. Lung Tumors Immunohistochemical staining of lung sections showing A) SPC (left panels) and PanCK (red) and DAPI (blue) (right panels) and B) HNF4A (red) and DAPI (blue) for different genetic backgrounds. Sections include SNL, SNL-Trp53, SNL-Pten, and LN2A, with tumor regions (T) and adjacent lung (L) regions indicated. SPC staining highlights surfactant protein C expression, and PanCK/DAPI illustrates epithelial marker PanCK with nuclear counterstaining using DAPI. Scale bar = 300 μm.

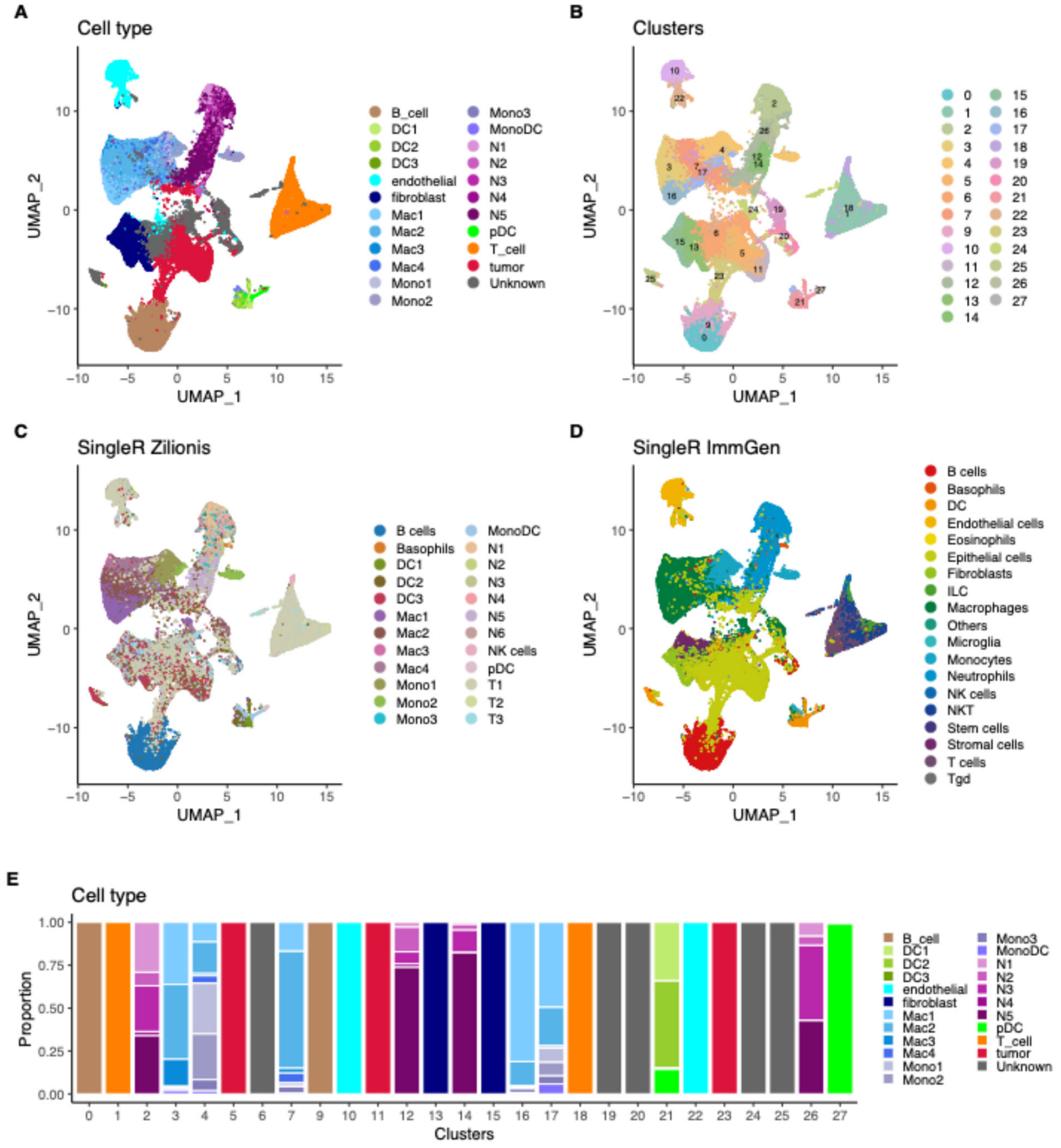

Supplementary Figure 6: Cell Type UMAP Annotations. UMAP of all cells (n=58,292). A) UMAP of cells colored by final cell type label. B) UMAP of all cells color by Louvain2 clusters at 0.8 resolution. C) UMAP of cells called by Single R with the Zilionis reference (Zillionis et al. 2019). D) UMAP of all cells called by SingleR with the ImmGen reference (Aran et al. 2019). E) Barplot of Louvain2 clusters, filled with the proportion of cell types by group.

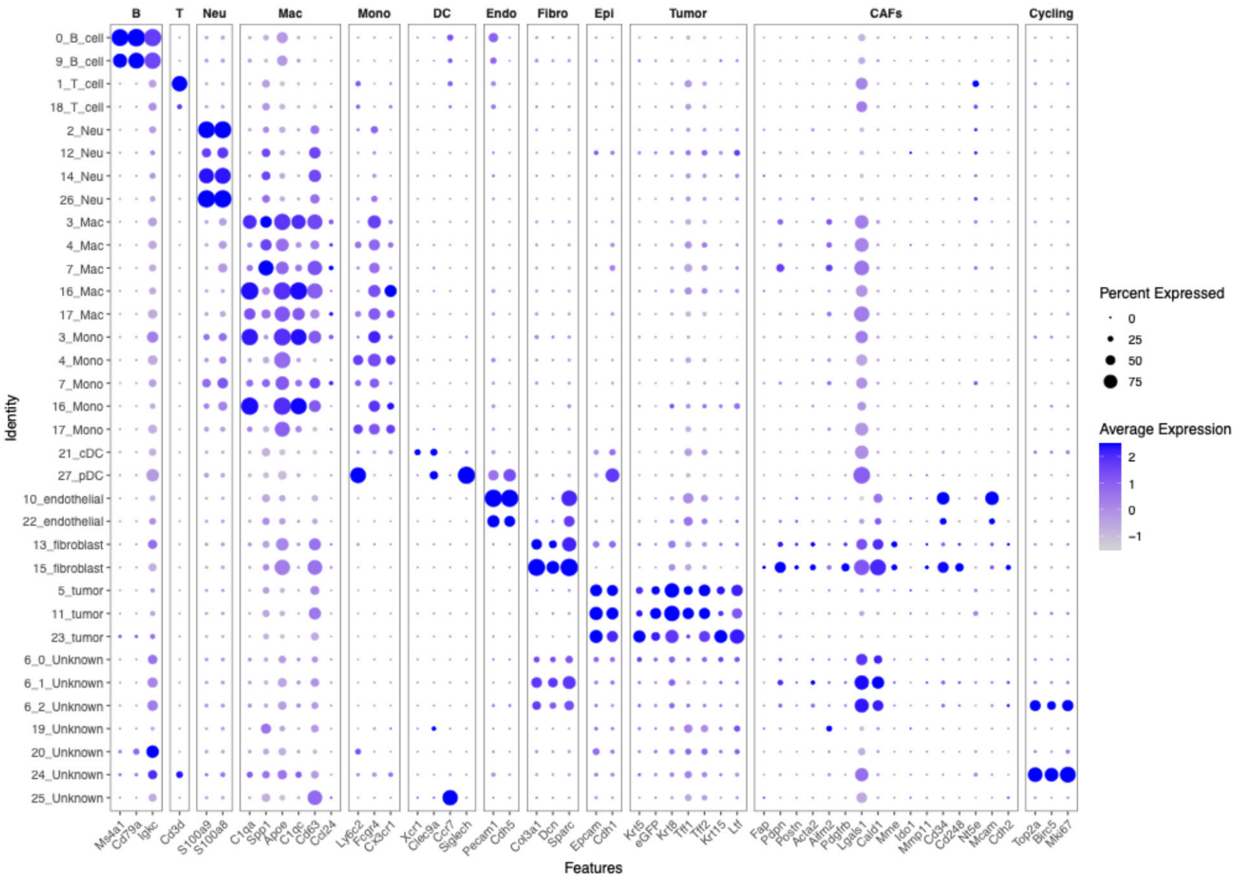

Supplementary Figure 7. Dotplot of marker gene expression across clusters and cell types. Cell type subtypes for neutrophils, macrophages, monocytes, and conventional dendritic cells are collapsed to major cell type by cluster. Abbreviations at the top of the figure indicate major cell types: B = B cells, T = T cells, Neu = neutrophils, Mac = macrophage, Mono = monocyte, DC = dendritic cell, Endo = endothelial cell, Fibro = fibroblasts, Epi = epithelial cells, CAFs = cancer-associated fibroblasts. CAF markers are derived from Mathieson et al. 2024 (https://www.nature.com/articles/s41416-024-02671-1), Cords et al. 2024 (https://www.cell.com/cancer-cell/fulltext/S1535-6108(23)00449-X), and Wu et al. 2024 (https://www.frontiersin.org/journals/immunology/articles/10.3389/fimmu.2024.1275064/full). Expression values are scaled.

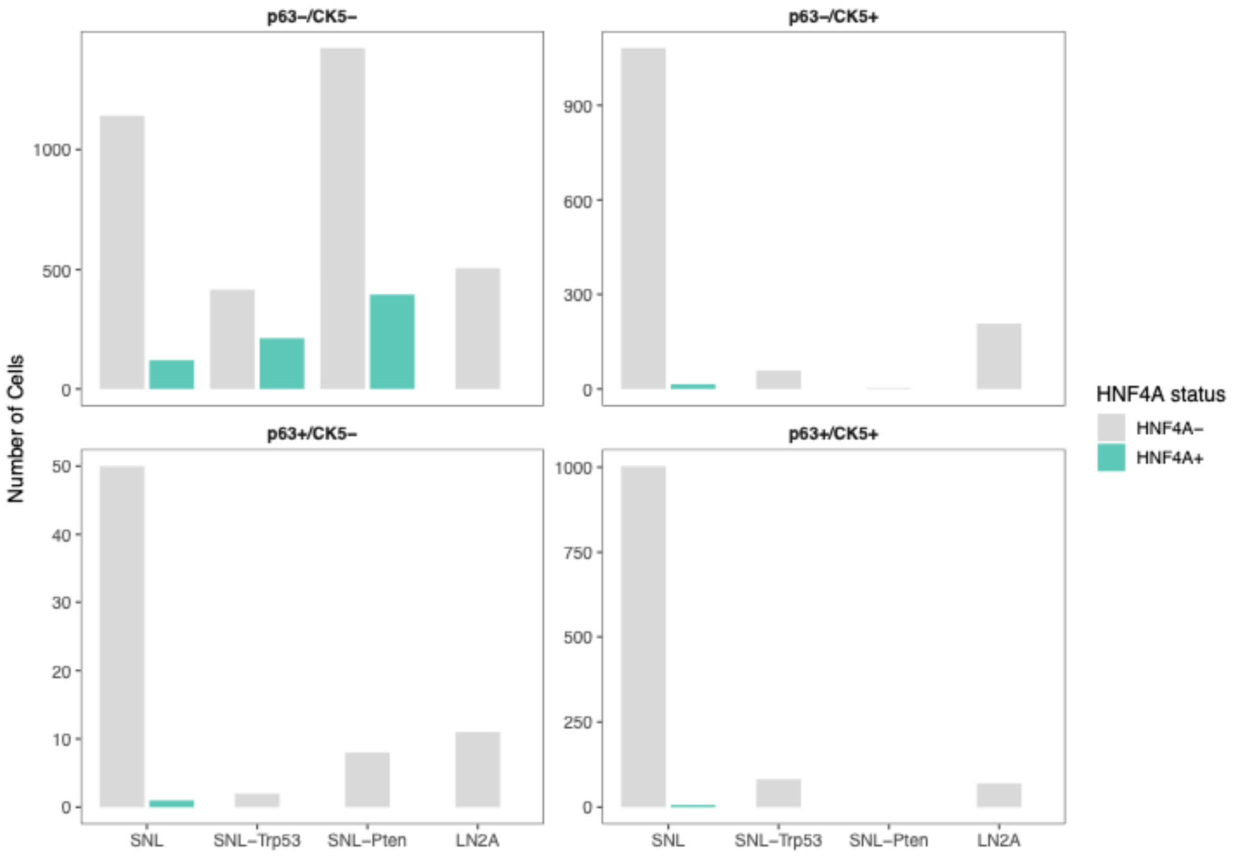

Supplementary Figure 8: HNF4A expression in tumor cells based on presence of CK5 and p63 by group. Barplot showing the number of *Hnf4a*+ cells for each combination of status for *CK5/p63*. A cell is considered positive for a gene, e.g., *CK5* if the log normalized expression is greater than 0.

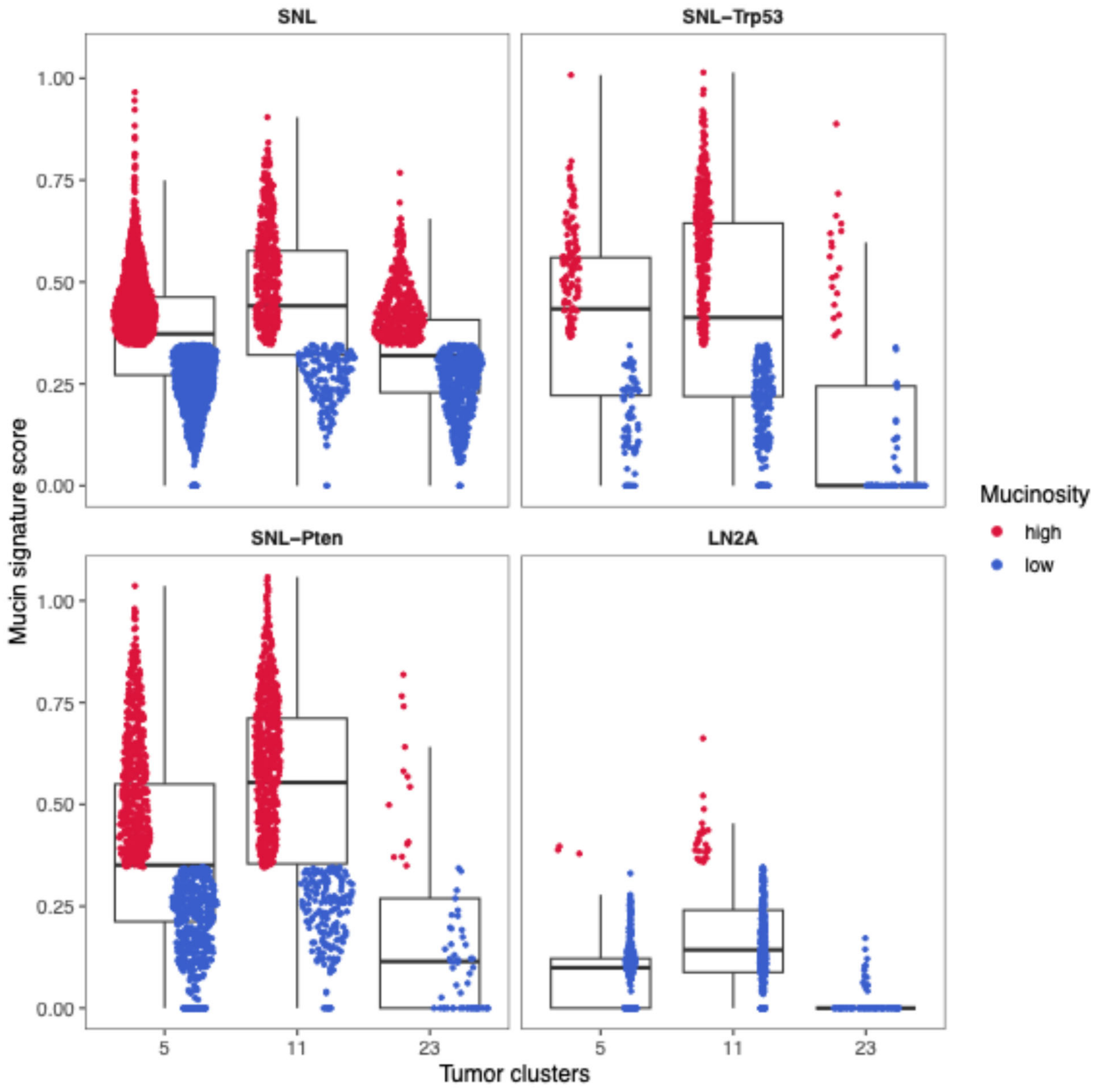

Supplementary Figure 9: Tumor Cell Mucin Clustering. Tumor cell clusters (clusters 5, 11 and 13) were all aligned with a previously published mucin differentiation signature (19). Cells above a mucin signature score of 0.5 were counted as mucin high (red). The gene signature used to define mucin low and high included the following genes: *Adam28, Adam8, Adamdec1, Agr2, Atp10b, Bcas1, Cldn2, Cp, Egln3, Elf3, Fermt1, Fut2, Hnf4a, Krt19, Lad1, Muc3, Muc5ac, Muc5b, Pax9, Plek2, Slc28a3, Slc44a4, Srd5a1, Tns4, Vtcn1 (19)*.

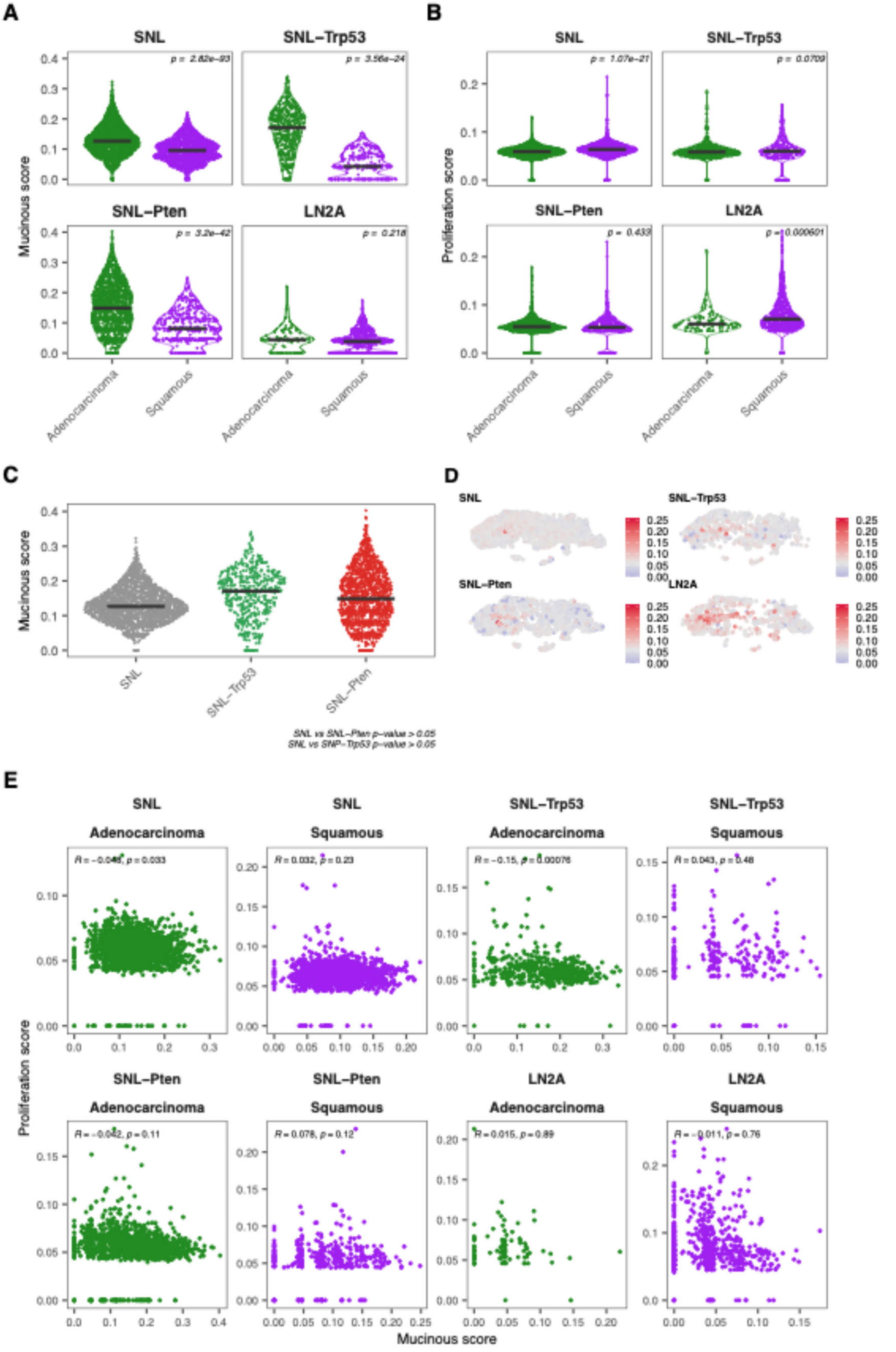
A
SNL
SNL−Trp53
SNL−Pten
LN2A
Mucinous score
p = 2.62e−93
p = 3.56e−24
p = 3.2e−42
p = 0.218
Adenocarcinoma
Squamous
B
SNL
SNL−Trp53
SNL−Pten
LN2A
Proliferation score
p = 1.07e−21
p = 0.0709
p = 0.433
p = 0.000601
Adenocarcinoma
Squamous
C
Mucinous score
SNL
SNL−Trp53
SNL−Pten
SNL vs SNL−Pten p−value > 0.05
SNL vs SNP−Trp53 p−value > 0.05
D
SNL
SNL−Trp53
SNL−Pten
LN2A
E
SNL Adenocarcinoma
SNL Squamous
SNL−Trp53 Adenocarcinoma
SNL−Trp53 Squamous
SNL−Pten Adenocarcinoma
SNL−Pten Squamous
LN2A Adenocarcinoma
LN2A Squamous
R = −0.046, p = 0.033
R = 0.032, p = 0.23
R = −0.15, p = 0.00076
R = 0.043, p = 0.48
R = −0.042, p = 0.11
R = 0.078, p = 0.12
R = 0.015, p = 0.89
R = −0.011, p = 0.76
Proliferation score
Mucinous score

Supplementary Figure 10: Mucinous signature in tumor cells. A) Violin and dotplot of mucinous signature score for each tumor model, colored by adenocarcinoma versus squamous categorization. Linear mixed effects models were used to test for differences in mucin score in adenocarcinoma versus squamous cells, with sample as a random effect. Tests were performed separately for each tumor model. P-values were approximated via Satterwhaite's method. Horizontal bars indicate the median value. B) Same as in A, with proliferation score. C) Mucinous signature score in adenocarcinoma cells by SNL model. Linear mixed effects models were used to test for differences in mucinous score. D) UMAP of tumor cells by tumor model (n=6,808 cells), colored by proliferation score. E) Mucinous and proliferation score for each tumor model, split by tumor cell classification (Adenocarcinoma versus Squamous). Spearman's correlation coefficient is shown for each facet, p-values are un-adjusted.

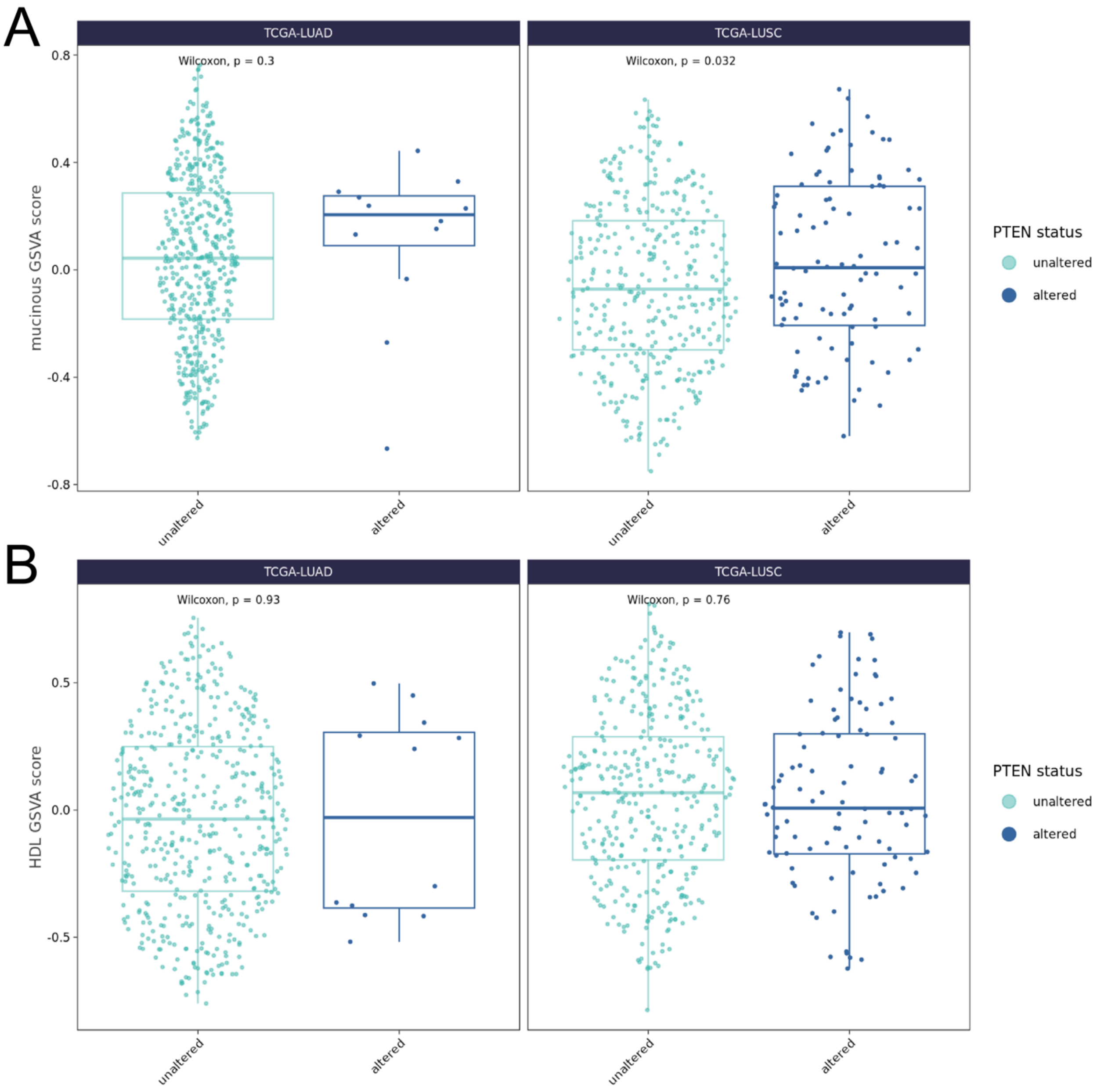

Supplementary Figure 11: Pten correlation with mucinous and HDL scores. A) Box plots showing the GSVA score for the mucinous gene set, facetted by TCGA data set, colored by *PTEN* status. A Kruskal-Wallis test was performed to test for differences in the mean score across all *PTEN* status groups shown. B) Box plots showing the GSVA score for the Kegg Reactome HDL Assembly gene set, facetted by TCGA data set, colored by *PTEN* alteration status. A Wilcoxon rank sum test was performed to test for differences in the mean score between samples that had any alteration (homozygous deletion or mutation) in *PTEN* versus those that did not.

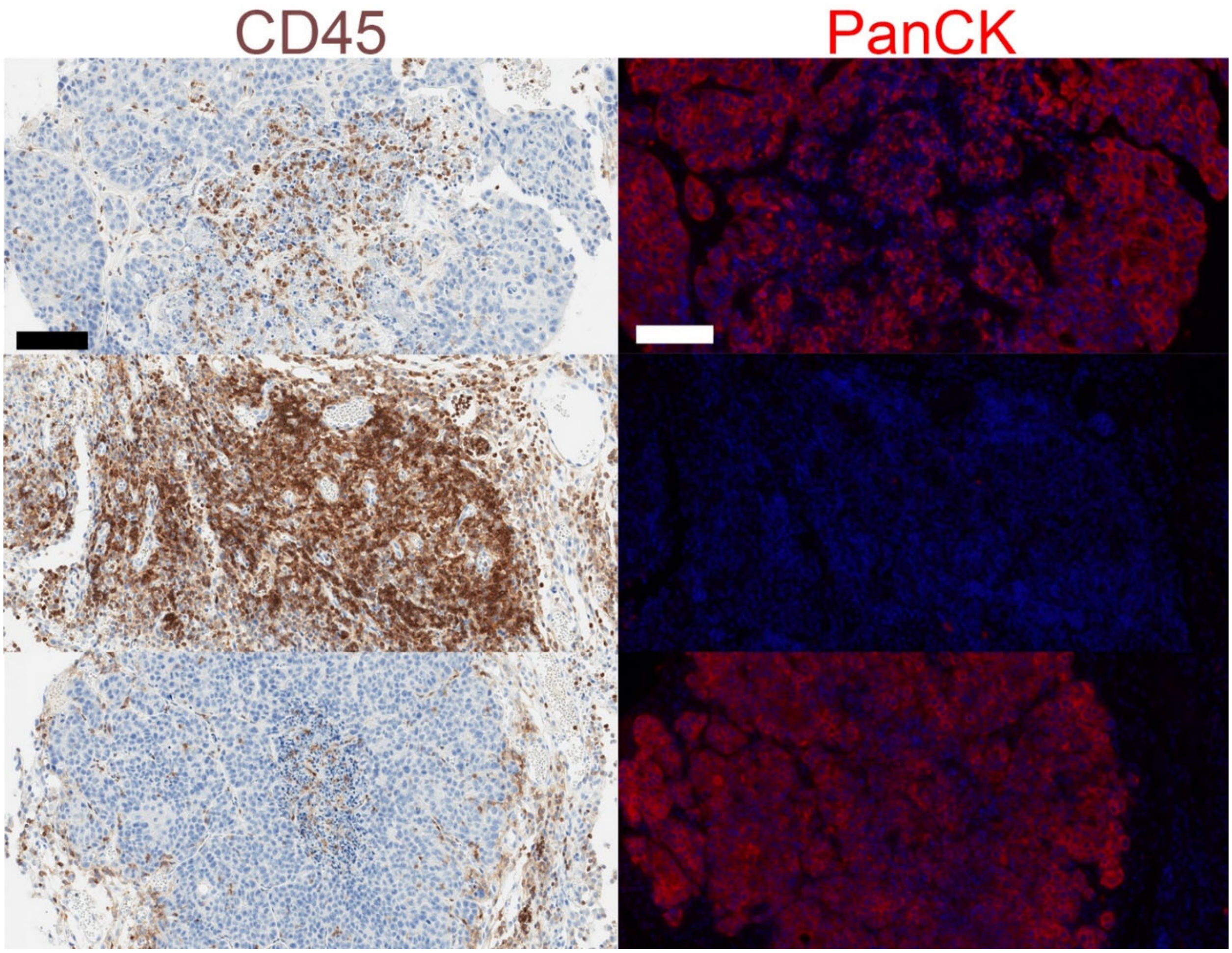

Supplementary Figure 12: LN2A CD45 and PanCK expression. All panels from different regions in the LN2A model with varying proportions of immune infiltrates. Left panel: CD45 single IHC DAB staining. Right panel: PanCK is shown in red immunofluorescent staining with a nuclear DAPI stain in blue. Scale bars = 100μM.

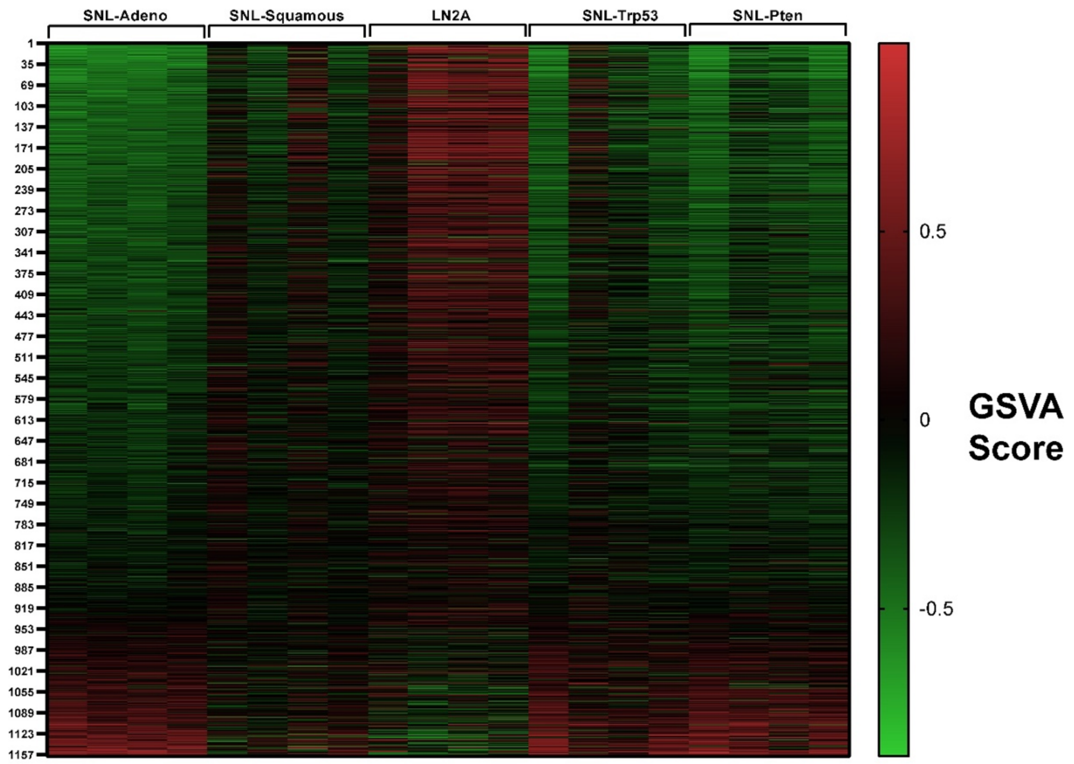

Supplementary Figure 13: GSVA alignment of Squamous and Adenocarcinoma. Pseudo-bulk data showing gene expression levels in the different tumor models (LN2A, SNL, SNL-Trp53 and SNL-Pten). Number aligns with gene-set shown in Supplementary Table 10. SNL models align with SNL-Adeno while LN2A aligns with SNL-Squamous.

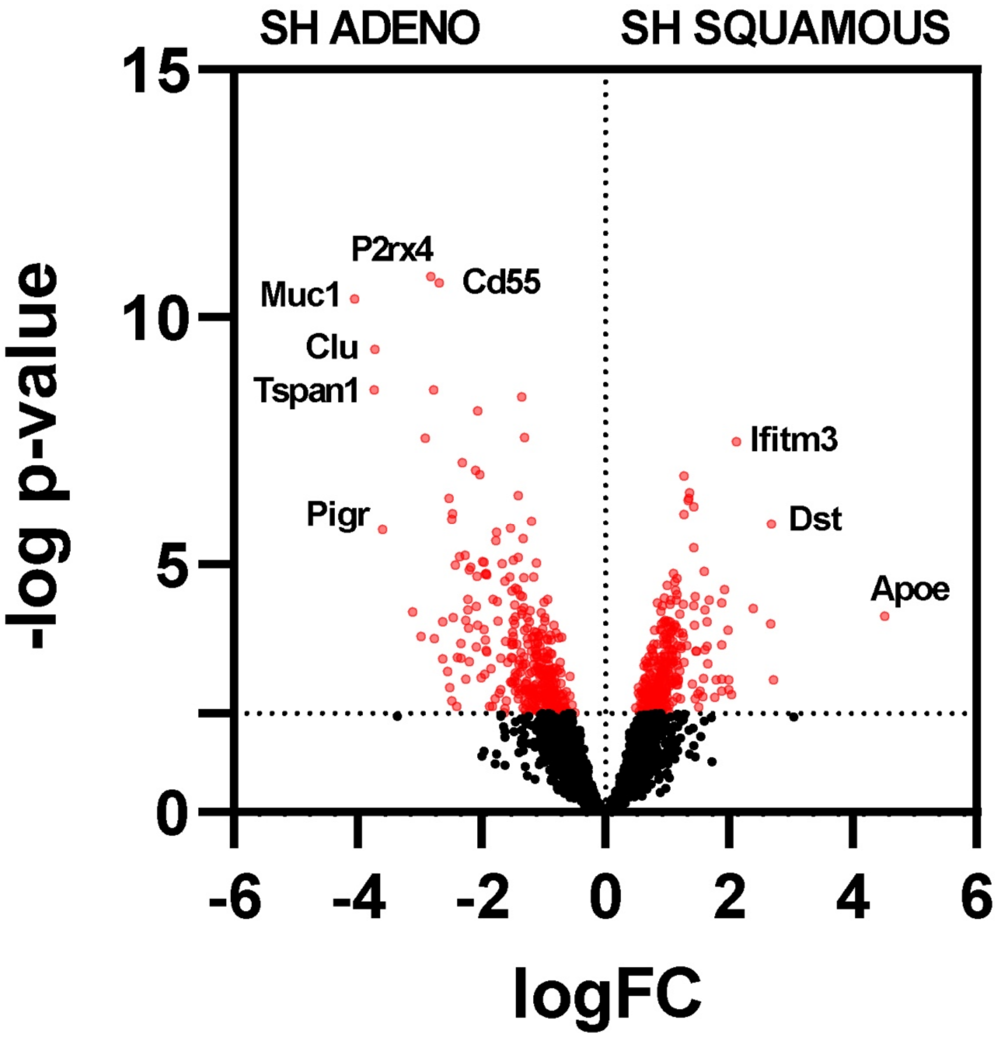

Supplementary Figure 14: Squamous vs Adenocarcinoma DEGs. Cells in the SNL models were labeled as Adeno or Squamous based off signature alignment to previously published work (Relli et al.). Gene expression changes between the two SNL models were captured by evaluating the differentially expressed genes. Red dots= significant p-value.

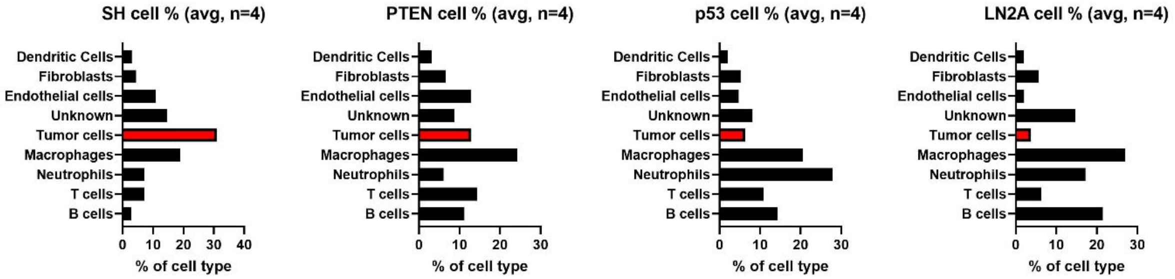

Supplementary Figure 15: General Populations. Distribution of various general cell populations across the different tumor models in order of increasing metastatic potential (SH, SNL-Pten, SNL-Trp53, LN2A) by scRNA-seq. Red=tumor cells.

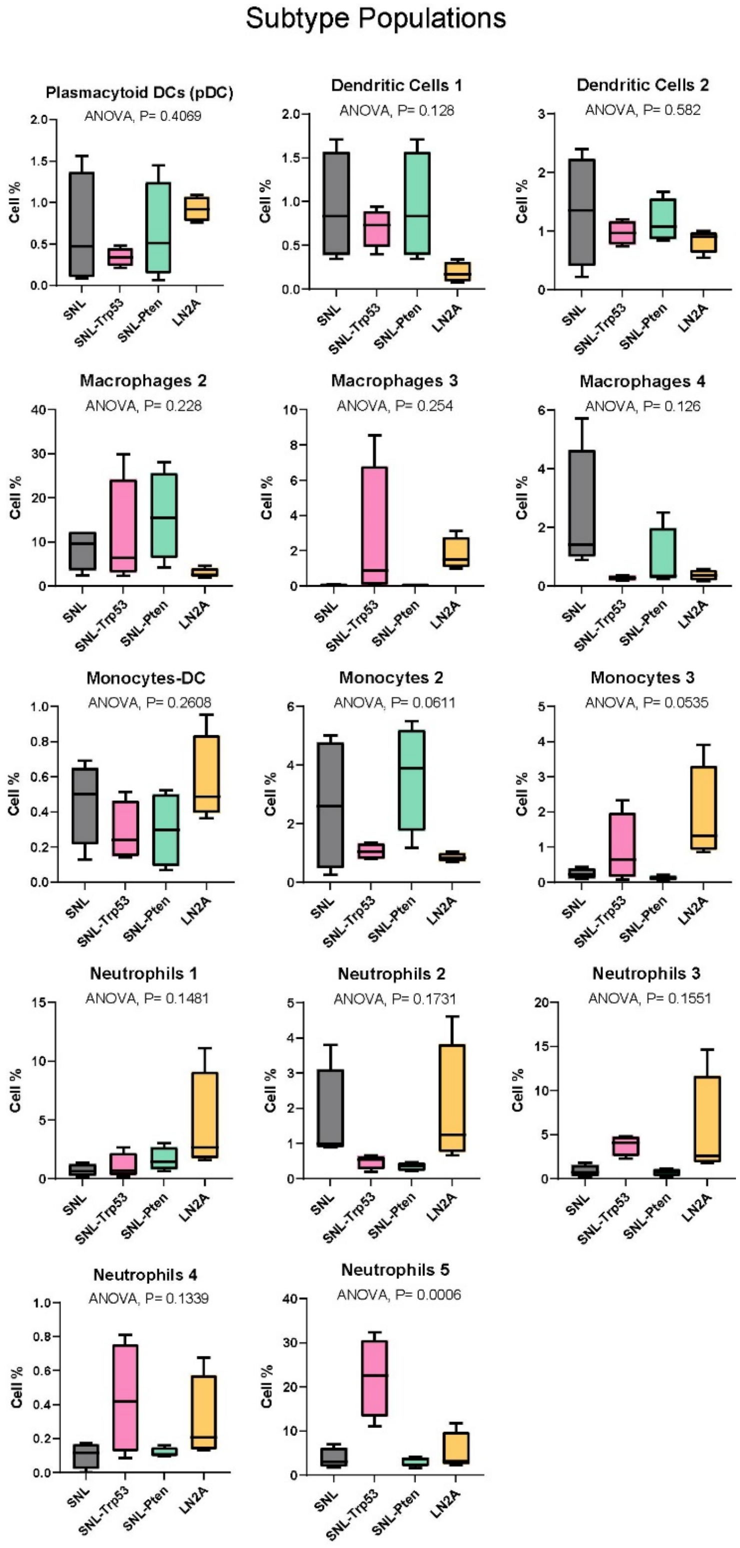

Supplementary Figure 16: Immunologic Infiltrates. Distribution of immune cell populations across the different tumor models (LN2A, SNL, SNL-Trp53 and SNL-Pten) by scRNA-seq.

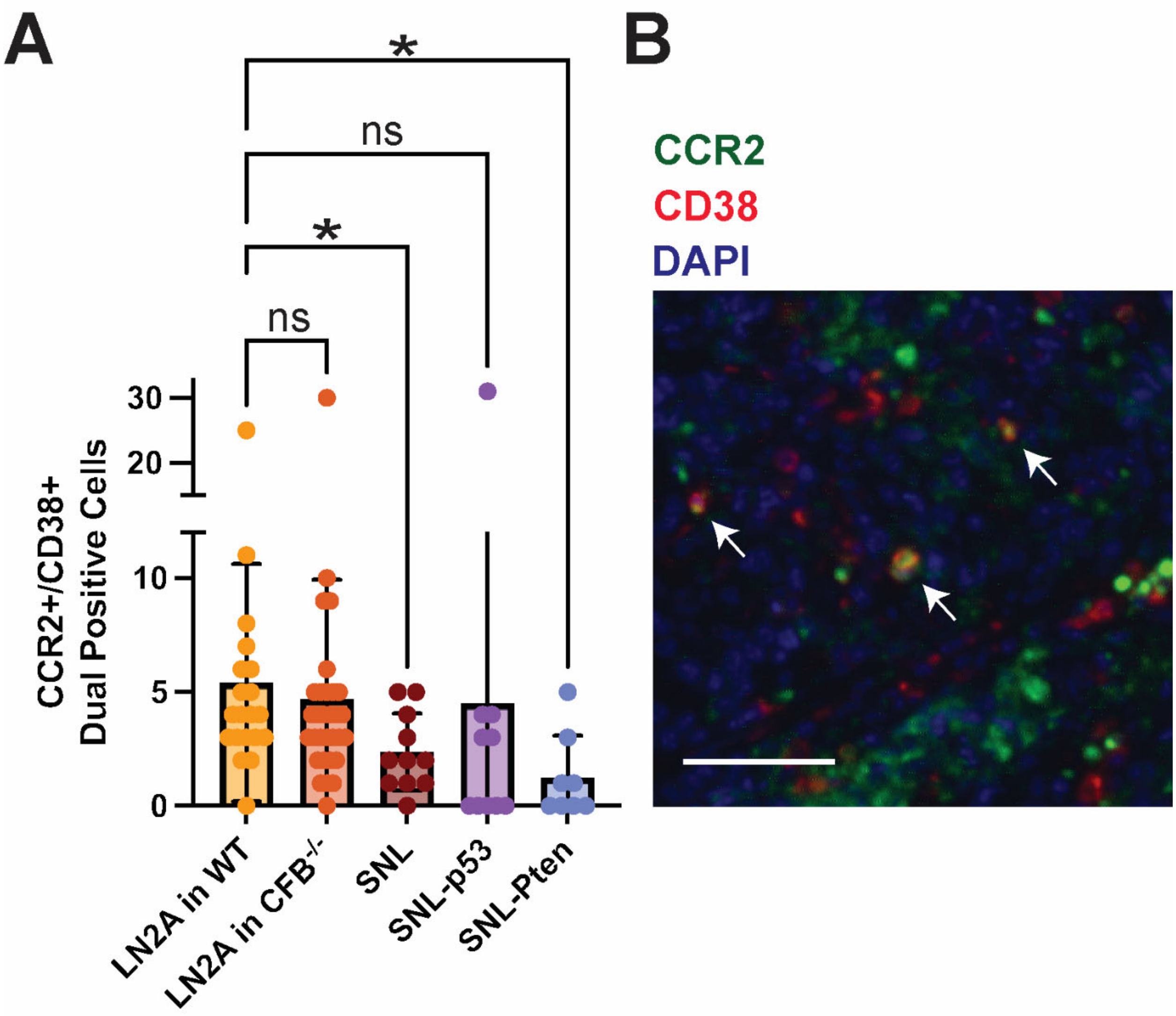

Supplementary Figure 17: Quantification and Visualization of CCR2+/CD38+ Dual-Positive Cells Across Tumor Models. A) Quantification of CCR2+/CD38+ dual-positive cells in LN2A tumors from wild-type (WT) mice, LN2A tumors in CFB-deficient (*Cfb*-/-) mice, and SNL, SNL-p53, and SNL-Pten models. A Kruskal-Wallis test was performed, yielding $p = 0.0027$. Further unpaired one-tailed t-tests comparing LN2A WT to other groups provided p-values: LN2A WT vs. LN2A in *Cfb*-/- ($p = 0.3159$), LN2A WT vs. SNL ($p = 0.0359$), LN2A WT vs. SNL-p53 ($p = 0.3689$), and LN2A WT vs. SNL-Pten ($p = 0.0192$). n=8-30 images analyzed per group. B) Representative immunofluorescence image showing CCR2 (green), CD38 (red), and DAPI (blue) co-localized (white arrows) in LN2A tumors. Scale bar represents 50 μm.

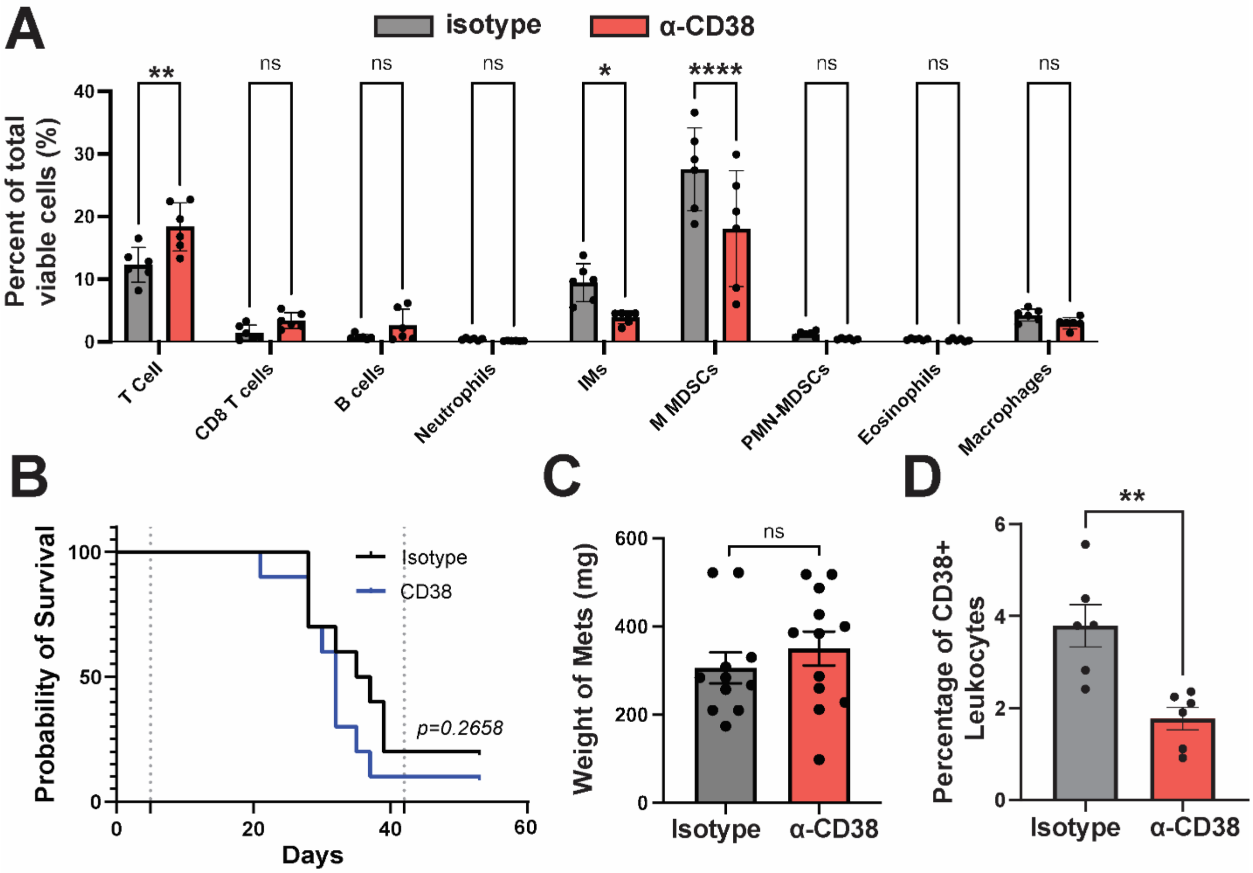

Supplementary Figure 18: Effect of CD38 Depletion in the LN2A model. A) Percentage of total leukocyte subtypes (T cells, CD8+ T cells, B cells, neutrophils, inflammatory monocytes (IMs), monocytic myeloid-derived suppressor cells (m-MDSCs), polymorphonuclear myeloid-derived suppressor cells (PMN-MDSCs), eosinophils, and macrophages) in mice treated with isotype control or α-CD38. Statistical significance was analyzed using Šídák's multiple comparisons test. Significant differences were observed in T cells ($p=0.0094$), IMs ($p=0.0239$), and M-MDSCs ($p<0.0001$), while other comparisons were not statistically significant (ns). Data are shown as mean ± SEM. B) Kaplan-Meier survival curve comparing mice treated with isotype control and α-CD38. Survival was analyzed using the log-rank (Mantel-Cox) test, with a p-value of 0.2658, n=10 per group. C) Metastatic weight (mg) in mice treated with isotype control or α-CD38. Data represent mean ± SEM, with individual data points shown for each mouse. Statistical significance was assessed using an unpaired t-test, $p=0.4076$, n=12 per group. D) Percentage of CD38+ cells within the CD45+ cell population in mice treated with either isotype control or α-CD38. Data represent mean ± SEM, with individual data points shown for each mouse. Statistical significance was assessed using an unpaired t-test, $p=0.0016$, n=6 per group.

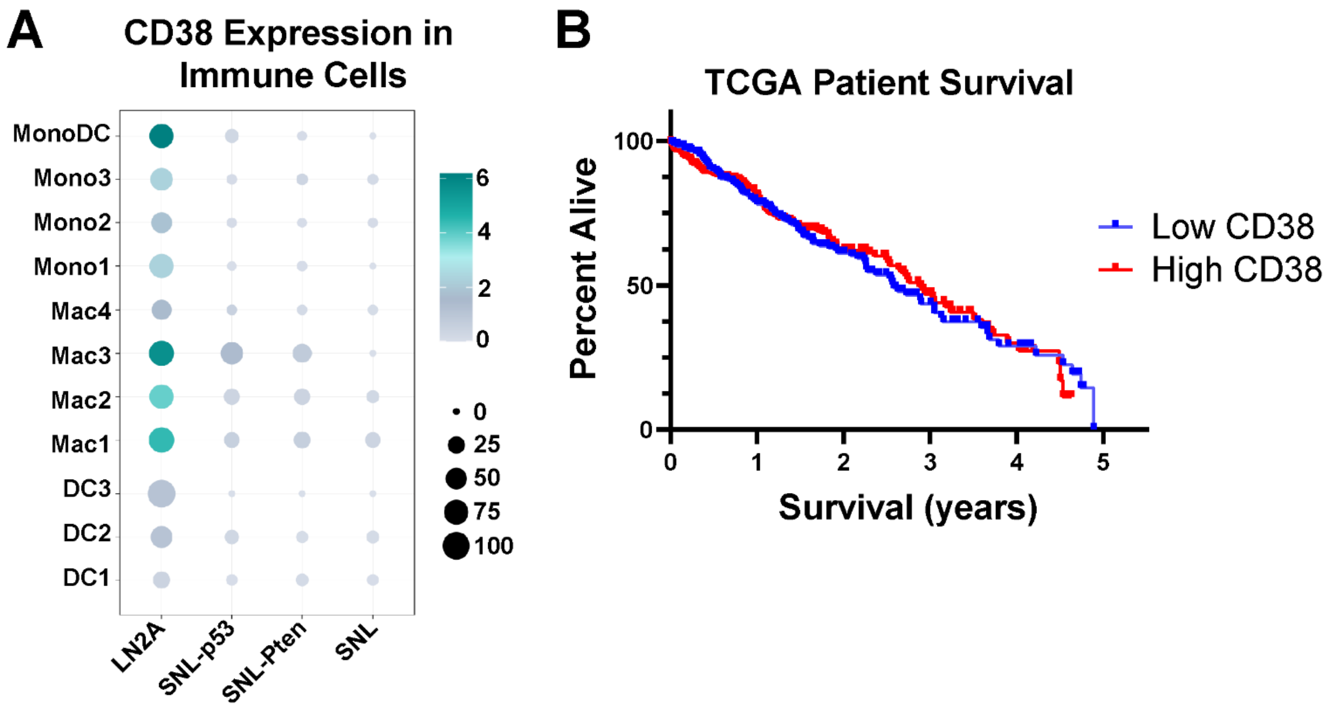

Supplementary Figure 19: CD38 expression patient survival and immune cell subset expression. A) Dot plot of CD38 expression across different immune cell subsets, including monocytes (Mono1-3), macrophages (Mac1-4), dendritic cells (DC1-3), and monocyte-derived dendritic cells (MonoDC). The color gradient indicates the level of CD38 expression, while the size of the dots represents the relative abundance of each cell type in different conditions (LN2A, SNL-p53, SNL-Pten, and SNL). B) Kaplan-Meier survival curve of TCGA patients grouped by CD38 expression levels (Low CD38 vs. High CD38 with a cutoff at the median). Survival is measured in years, and differences between groups are visualized over a 5-year period.

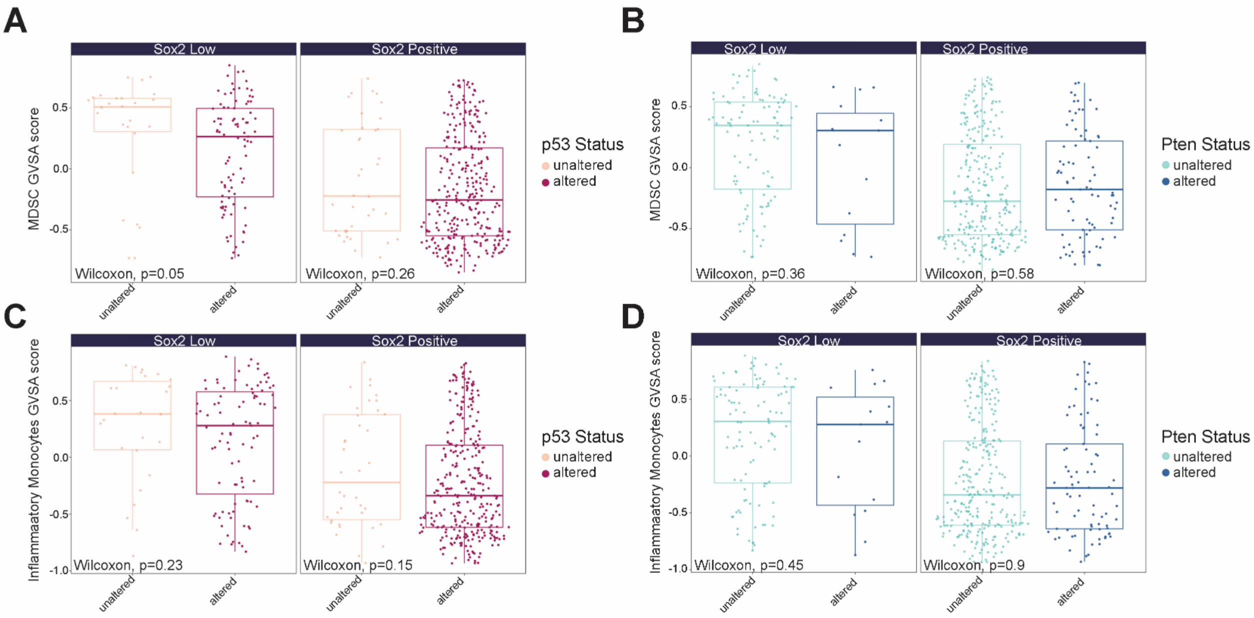

Supplementary Figure 20: Association of MDSC Signatures in human SOX2 high and low TCGA-LUSC samples. A) MDSC GSVA scores in samples categorized as *SOX2* Low (below the first quartile) and *SOX2* Positive (at or above the first quartile), further divided by P*53* status (unaltered vs. altered). B) MDSC GSVA scores in *SOX2* Low and *SOX2* Positive samples based on *PTEN* status. Differences were analyzed using Wilcoxon rank-sum tests for each *SOX2* expression level. C) Inflammatory monocyte GSVA scores in *SOX2* Low and *SOX2* Positive samples based on *P53* status. D) Inflammatory monocyte GSVA scores in *SOX2* Low and *SOX2* Positive samples based on *PTEN* status. Differences were analyzed using Wilcoxon rank-sum tests for each *SOX2* expression level.

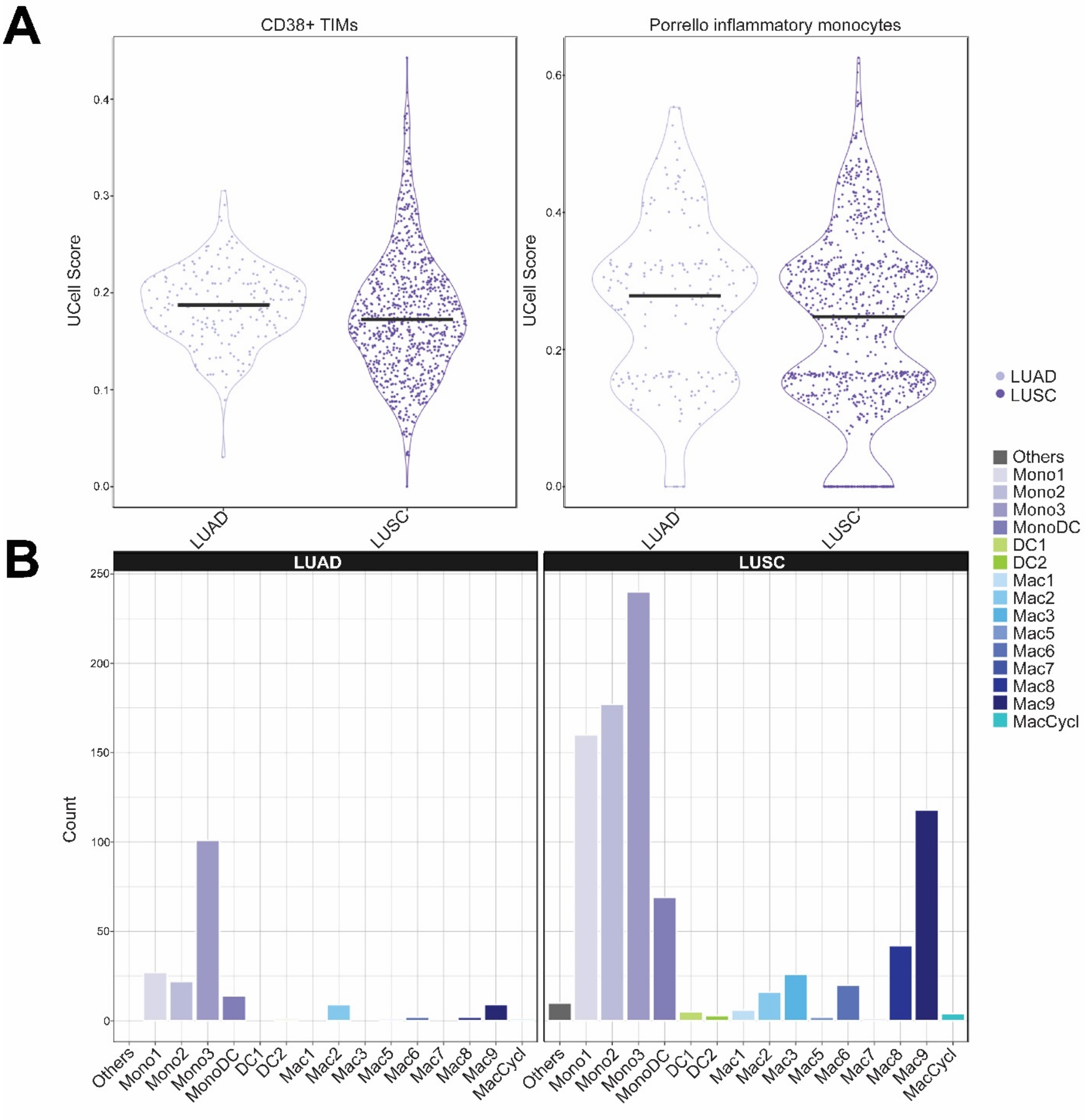

Supplementary Figure 21: Comparison of Inflammatory Monocyte Signatures and Composition in Human LUAD and LUSC. A) Violin plots display UCell signature scores for inflammatory monocytes classified by CD38+ TIMs and Porrello IMs across TCGA data from LUAD (lung adenocarcinoma) and LUSC (lung squamous cell carcinoma). The scores represent inflammatory signatures in myeloid subsets, as calculated through UCell. B) Bar plots represent cell type counts annotated from single-cell RNA-Seq data of LUAD and LUSC clinical samples. Myeloid cell subsets were identified following clustering and marker gene analysis. Cell types include various macrophages (Mac1-Mac9, MacCycl), monocytes (Mono1-Mono3), and dendritic cells (DC1, DC2). Counts were stratified by cancer type, highlighting distinct myeloid composition in LUAD vs. LUSC.

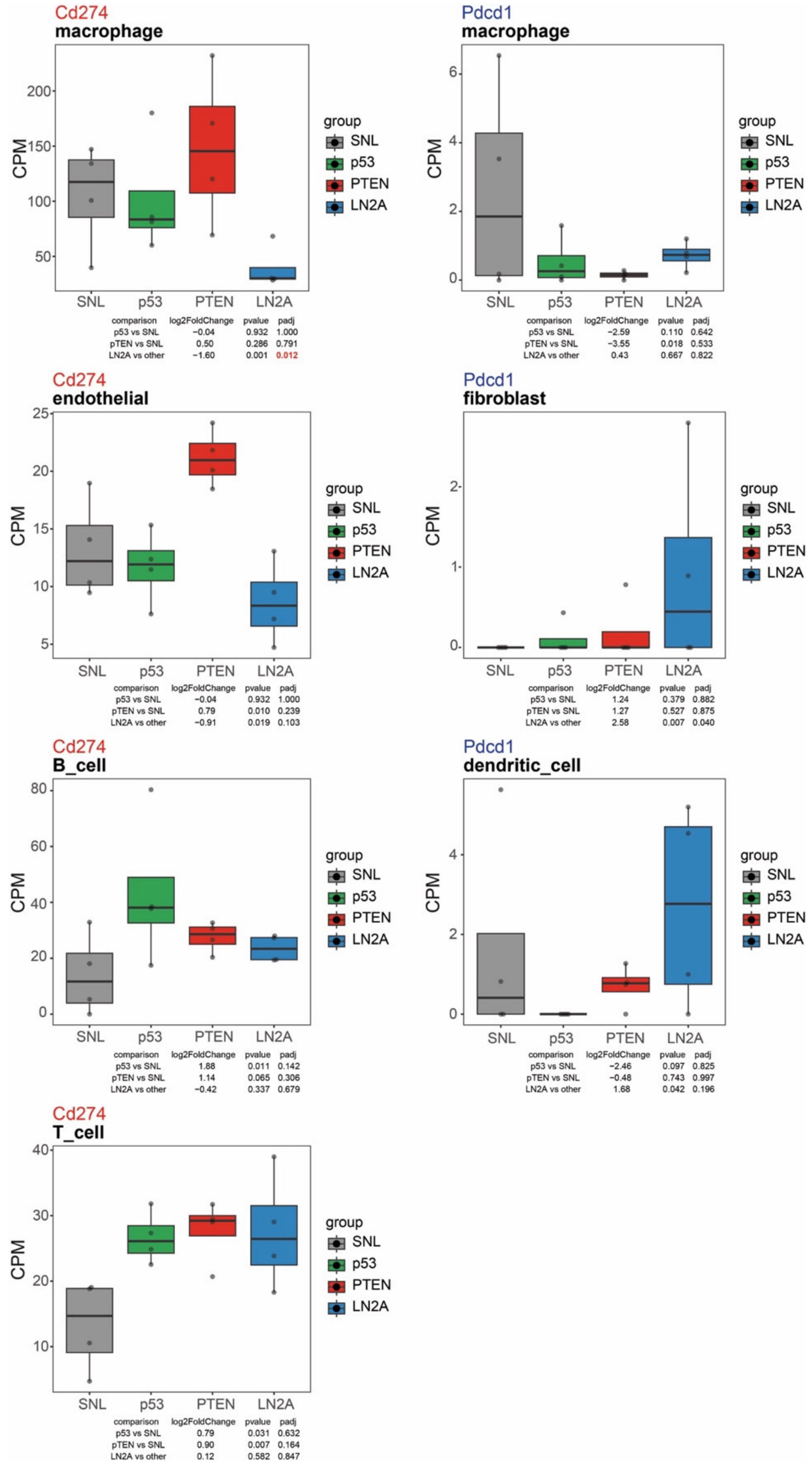

Supplementary Figure 22. PD/PDL1 differential expression analysis. Differential expression analysis for PDL1 (*Cd274*) and PD1 (*Pdcd1*) was conducted via the pseudo-bulk method on scRNA-Seq data, with raw cell counts aggregated at the sample level. All p-values were adjusted for multiple hypothesis testing utilizing the Benjamini-Hochberg (FDR) method.

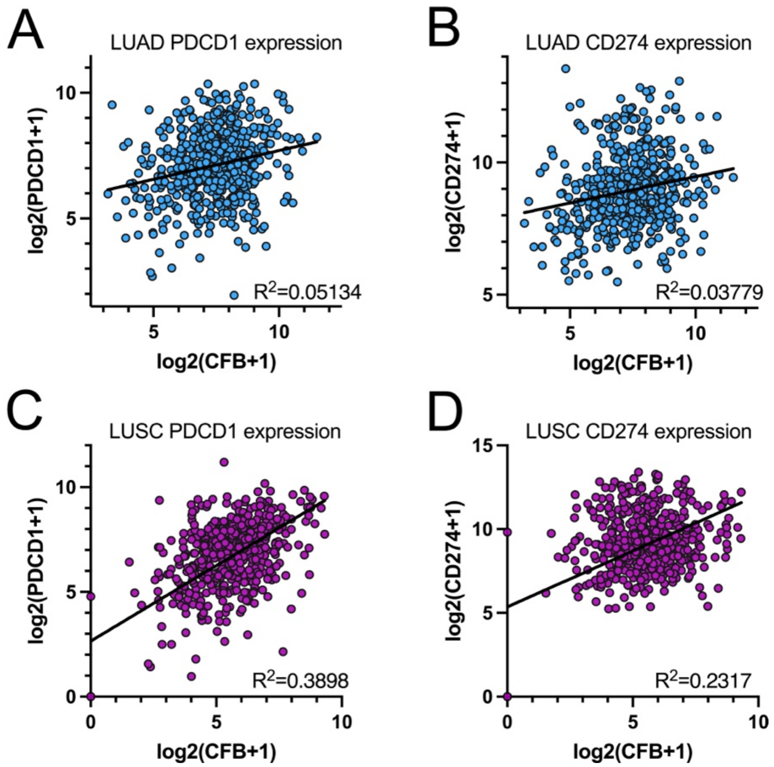

Supplementary Figure 23. CFB correlation with PDL1 and PD1 in LUSC and LUAD TCGA data. A) Scatterplot depicting the correlation between *CFB* expression (log2[CFB+1]) and *PDCD1* expression (log2[PDCD1+1]) in LUAD, with an R^2 value of 0.05134, Equation: “Y = 0.2287*X + 5.416”, p<0.0001. B) Scatterplot showing the correlation between *CFB* expression (log2[CFB+1]) and *CD274* expression (log2[CD274+1]) in LUAD, with an R^2 value of 0.03779, Equation: "Y = 0.1994*X + 7.471", p<0.0001. C) Scatterplot demonstrating the correlation between *CFB* expression (log2[CFB+1]) and *PDCD1* expression (log2[PDCD1+1]) in LUSC, with an R^2 value of 0.3886, Equation: "Y = 0.7208*X + 2.649", p<0.0001. D) Scatterplot illustrating the correlation between *CFB* expression (log2[CFB+1]) and *CD274* expression (log2[CD274+1]) in LUSC, with an R^2 value of 0.2317, Equation: "Y = 0.6682*X + 5.356" p<0.0001.

REFERENCES

1. Hao Y, Hao S, Andersen-Nissen E, Mauck WM, Zheng S, Butler A, et al. Integrated analysis of multimodal single-cell data. Cell. 2021;184(13):3573-87.e29.
2. McCarthy DJ, Campbell KR, Lun AT, Wills QF. Scater: pre-processing, quality control, normalization and visualization of single-cell RNA-seq data in R. Bioinformatics. 2017;33(8):1179-86.
3. Germain PL, Lun A, Garcia Meixide C, Macnair W, Robinson MD. Doublet identification in single-cell sequencing data using. F1000Res. 2021;10:979.
4. Hafemeister C, Satija R. Normalization and variance stabilization of single-cell RNA-seq data using regularized negative binomial regression. Genome Biol. 2019;20(1):296.
5. McInnes L, Healy Jo, Melville J. UMAP: Uniform Manifold Approximation and Projection for Dimension Reduction. Cornell arXiv2020.
6. Zilionis R, Engblom C, Pfirschke C, Savova V, Zemmour D, Saatcioglu HD, et al. Single-Cell Transcriptomics of Human and Mouse Lung Cancers Reveals Conserved Myeloid Populations across Individuals and Species. Immunity. 2019;50(5):1317-34.e10.
7. Aran D, Looney AP, Liu L, Wu E, Fong V, Hsu A, et al. Reference-based analysis of lung single-cell sequencing reveals a transitional profibrotic macrophage. Nat Immunol. 2019;20(2):163-72.
8. Heng TSP, Painter MW, Elpek K, Lukacs-Kornek V, Mauermann N, Turley SJ, et al. The Immunological Genome Project: networks of gene expression in immune cells. Nature Immunology. 2008;9(10):1091-4.
9. Korsunsky I, Millard N, Fan J, Slowikowski K, Zhang F, Wei K, et al. Fast, sensitive and accurate integration of single-cell data with Harmony. Nat Methods. 2019;16(12):1289-96.
10. Waltman L, Van Eck NJ. A smart local moving algorithm for large-scale modularity-based community detection. The European Physical Journal B. 2013;86(11).
11. Robinson MD, McCarthy DJ, Smyth GK. edgeR: a Bioconductor package for differential expression analysis of digital gene expression data. Bioinformatics. 2010;26(1):139-40.
12. McCarthy DJ, Chen Y, Smyth GK. Differential expression analysis of multifactor RNA-Seq experiments with respect to biological variation. Nucleic Acids Res. 2012;40(10):4288-97.
13. Chen Y, Lun AT, Smyth GK. From reads to genes to pathways: differential expression analysis of RNA-Seq experiments using Rsubread and the edgeR quasi-likelihood pipeline. F1000Res. 2016;5:1438.
14. Subramanian A, Tamayo P, Mootha VK, Mukherjee S, Ebert BL, Gillette MA, et al. Gene set enrichment analysis: a knowledge-based approach for interpreting genome-wide expression profiles. Proc Natl Acad Sci U S A. 2005;102(43):15545-50.
15. Liberzon A, Birger C, Thorvaldsdóttir H, Ghandi M, Mesirov JP, Tamayo P. The Molecular Signatures Database (MSigDB) hallmark gene set collection. Cell Syst. 2015;1(6):417-25.
16. Hänzelmann S, Castelo R, Guinney J. GSVA: gene set variation analysis for microarray and RNA-Seq data. BMC Bioinformatics. 2013;14(1):7.
17. Ritchie ME, Phipson B, Wu D, Hu Y, Law CW, Shi W, et al. limma powers differential expression analyses for RNA-sequencing and microarray studies. Nucleic Acids Res. 2015;43(7):e47.
18. Andreatta M, Carmona SJ. UCell: Robust and scalable single-cell gene signature scoring. Comput Struct Biotechnol J. 2021;19:3796-8.
19. Guo M, Tomoshige K, Meister M, Muley T, Fukazawa T, Tsuchiya T, et al. Gene signature driving invasive mucinous adenocarcinoma of the lung. EMBO Mol Med. 2017;9(4):462-81.
20. Wu F, Fan J, He Y, Xiong A, Yu J, Li Y, et al. Single-cell profiling of tumor heterogeneity and the microenvironment in advanced non-small cell lung cancer. Nature communications. 2021;12(1):2540.
21. https://www.ncbi.nlm.nih.gov/datasets/genome/GCF_000001635.26/ Gpg.

22. Dobin A, Davis CA, Schlesinger F, Drenkow J, Zaleski C, Jha S, et al. STAR: ultrafast universal RNA-seq aligner. Bioinformatics. 2013;29(1):15-21.
23. Patro R, Duggal G, Love MI, Irizarry RA, Kingsford C. Salmon provides fast and bias-aware quantification of transcript expression. Nat Methods. 2017;14(4):417-9.
24. Institute B. Picard Toolkit 2019 [Available from: https://broadinstitute.github.io/picard/.
25. FastQC: https://www.bioinformatics.babraham.ac.uk/projects/fastqc/.
26. Ewels P, Magnusson M, Lundin S, Käller M. MultiQC: summarize analysis results for multiple tools and samples in a single report. Bioinformatics. 2016;32(19):3047-8.
27. GENCODE vM25 transcriptome: https://www.gencodegenes.org/mouse/release_M25.html.
28. Korotkevich G, Sukhov V, Budin N, Shpak B, Artyomov MN, Sergushichev A. Fast gene set enrichment analysis. bioRxiv. 2021:060012.
29. Documentation NCI-G. GDC API calls [Available from: https://docs.gdc.cancer.gov/API/Users_Guide/Search_and_Retrieval/.
30. Love MI, Huber W, Anders S. Moderated estimation of fold change and dispersion for RNA-seq data with DESeq2. Genome Biol. 2014;15(12):550.
31. Cerami E, Gao J, Dogrusoz U, Gross BE, Sumer SO, Aksoy BA, et al. The cBio cancer genomics portal: an open platform for exploring multidimensional cancer genomics data. Cancer Discov. 2012;2(5):401-4.
32. Liberzon A, Subramanian A, Pinchback R, Thorvaldsdottir H, Tamayo P, Mesirov JP. Molecular signatures database (MSigDB) 3.0. Bioinformatics. 2011;27(12):1739-40.
33. Porrello A, Leslie PL, Harrison EB, Gorentla BK, Kattula S, Ghosh SK, et al. Factor XIIIA-expressing inflammatory monocytes promote lung squamous cancer through fibrin cross-linking. Nature communications. 2018;9(1):1988.
34. Terry M Therneau [aut c, Thomas Lumley [ctb, trl] (original S->R port and R maintainer until 2009), Atkinson Elizabeth [ctb], Crowson Cynthia [ctb]. survival: Survival Analysis 2024 [3.8-3:[Available from: https://cran.r-project.org/web/packages/survival/index.html.
35. Alboukadel Kassambara MK, Przemyslaw Biecek. survminer: Drawing Survival Curves using 'ggplot2' 2024 [R package version 0.5.0:[Available from: https://rpkgs.datanovia.com/survminer/index.html.
